# Reviewers in 2023

**DOI:** 10.1098/rspb.2024.0323

**Published:** 2024-03-27

**Authors:** Proceedings B Editorial Team

The Editors of *Proceedings B* thank all reviewers who contributed their time by refereeing manuscripts submitted throughout 2023. From previous feedback, we know that most reviewers value recognition of their efforts by the journal and through services, such as Web of Science Reviewer Recognition Service (previously Publons), which is integrated with *Proceedings B*.

To provide an opportunity for recognition, we list the names of all reviewers (unless they have opted out). This article is made permanent and citeable by its digital object identifier (DOI). We encourage you to quote your contribution in your applications for tenure and grants or other forms of research assessment. Where you have provided it, we have included your ORCID, so your contribution can be unambiguously assigned to you.

The team really does appreciate all the work our reviewers do on behalf of the journal, and we look forward to working with you again in 2024.

Feiner N https://orcid.org/0000-0003-4648-6950

Vilcinskas A https://orcid.org/0000-0001-8276-4968

Beik M

Beaulieu M https://orcid.org/0000-0002-9948-269X

Barve S https://orcid.org/0000-0001-5840-8023

Taubert J

Osváth G

Garriga P

Surbeck M

Hone D

Koprivnikar J https://orcid.org/0000-0001-8410-1041

Prochazka P https://orcid.org/0000-0001-9385-4547

Streinzer M

Seel C

Pita L

Chindelevitch L

Ruiz-Gonzalez M

Fauteux D https://orcid.org/0000-0001-5373-8701

Laskowski K https://orcid.org/0000-0003-1523-9340

Dias M

Solferini V

Leger L

Christie K https://orcid.org/0000-0001-8257-2106

Senior A https://orcid.org/0000-0001-9805-7280

Wagner M

Tidière M https://orcid.org/0000-0001-6339-5765

Rafaluk C

Gardiner J https://orcid.org/0000-0003-1902-3416

Bräuer J

Raymann K

Wright D https://orcid.org/0000-0003-2329-2635

Czaczkes T https://orcid.org/0000-0002-1350-4975

Huey R https://orcid.org/0000-0002-4962-8670

Mooers A

Starzomski B

Link A

Hopkins B https://orcid.org/0000-0002-9760-6185

Minamoto T

Assis B https://orcid.org/0000-0003-0054-6639

Patlar B https://orcid.org/0000-0002-0442-9061

Cleghorn N

Turner P

Grosjean P https://orcid.org/0000-0002-3623-8083

Iossa G https://orcid.org/0000-0001-6813-4361

Bath E https://orcid.org/0000-0002-2988-8061

Kallio E https://orcid.org/0000-0003-2991-612X

Huss M https://orcid.org/0000-0002-5131-6000

Maas M https://orcid.org/0000-0003-0122-106X

Whitehouse J https://orcid.org/0000-0003-2607-5492

Viana M https://orcid.org/0000-0001-5975-6505

Pincheira-Donoso D

Pirk C

Takahashi A

Caillaud D

Tsuboi M https://orcid.org/0000-0002-0144-2893

Storck-Tonon D

Haug J

Turnbull C

Firman R https://orcid.org/0000-0001-9428-7388

Mysterud A https://orcid.org/0000-0001-8993-7382

Beauchamp G https://orcid.org/0000-0002-3393-9562

Winkler L https://orcid.org/0000-0003-3597-6540

Saulnier-Talbot E

García-Roger E

Calabrese G https://orcid.org/0000-0002-9092-2396

Neate-Clegg M https://orcid.org/0000-0001-9753-6765

Modarres-Sadeghi Y

McCullough E https://orcid.org/0000-0002-6886-9445

Buckley M https://orcid.org/0000-0002-4166-8213

Romano V https://orcid.org/0000-0002-4128-9205

Green J

Schütz A

Wei C-L

Gomez Isaza D https://orcid.org/0000-0003-3112-8683

MacLean H https://orcid.org/0000-0003-0982-3209

Schweinfurth M https://orcid.org/0000-0003-2066-7892

Kiel S https://orcid.org/0000-0001-6281-100X

Lougheed S

Krupp DB https://orcid.org/0000-0001-6297-7739

Grundel R

Reindl E

Anderson J

McAuley T

Brückner A https://orcid.org/0000-0002-9184-8562

Foster W

Vale G

Mcandry C

Kagawa K https://orcid.org/0000-0002-5749-5679

Yannelli F

Johnson L

DeLong J https://orcid.org/0000-0003-0558-8213

Neate-Clegg M https://orcid.org/0000-0001-9753-6765

Young G

Simms E

Zanno L

Pelisson D

Hargrove J https://orcid.org/0000-0003-0346-5260

Albrecht J https://orcid.org/0000-0002-9708-9413

Jones W https://orcid.org/0000-0002-3058-0072

Cook J https://orcid.org/0000-0002-7320-1706

Preisser W https://orcid.org/0000-0002-4967-3930

Moore H

Fay R https://orcid.org/0000-0002-7202-367X

Wotton K https://orcid.org/0000-0002-8672-9948

Tschinkel W

Ashander J https://orcid.org/0000-0002-1841-4768

Smith D https://orcid.org/0000-0003-4367-6556

Lv L

Teitelbaum C https://orcid.org/0000-0001-5646-3184

Fish F

Reddy RB

Ives A https://orcid.org/0000-0001-9375-9523

McGuigan N

Cooper R https://orcid.org/0000-0001-7234-4361

Mudrik L

Morris M https://orcid.org/0000-0001-9962-2982

Somanathan H https://orcid.org/0000-0002-4803-3894

Putman N https://orcid.org/0000-0001-8485-7455

Ryabov EV

Venditti C https://orcid.org/0000-0002-6776-2355

Hulber K

Lewanzik D

Bessho K https://orcid.org/0000-0002-5911-6855

Shaffer S

Liu J https://orcid.org/0000-0002-1923-5964

Crandell K https://orcid.org/0000-0002-2150-3198

Collins A

Badás E

Streit R

Cohen S

Sattenspiel L https://orcid.org/0000-0003-4534-3392

Sørensen JG

Albery G https://orcid.org/0000-0001-6260-2662

Sims D

Mangiacotti M https://orcid.org/0000-0001-7144-3851

Tyrrell L

McMenamin A

MacNulty D

Drobniak S

Stien A https://orcid.org/0000-0001-8046-7337

Taylor E

Slate J https://orcid.org/0000-0003-3356-5123

Smith T

Schenkel M https://orcid.org/0000-0002-7721-319X

Hebets E https://orcid.org/0000-0002-9382-2040

Lisi M https://orcid.org/0000-0003-3554-385X

Moore A https://orcid.org/0000-0002-1498-3322

Dick C

Svensson E https://orcid.org/0000-0001-9006-016X

Singer M

Cueva del Castillo R https://orcid.org/0000-0002-6385-0729

Prugh L

Reuter D https://orcid.org/0000-0001-5637-7331

Ginot S https://orcid.org/0000-0003-0060-9660

Davis G https://orcid.org/0000-0003-3312-4942

Fu F

Williams C

Jasienska G https://orcid.org/0000-0001-8716-6342

Pilastro A https://orcid.org/0000-0001-9803-6308

Aanen D https://orcid.org/0000-0002-5702-1617

Arroyo-Machado W https://orcid.org/0000-0001-9437-8757

Sandkam B https://orcid.org/0000-0002-5043-9295

Jackson ST

Cuesta F https://orcid.org/0000-0002-5150-073X

Sneddon L https://orcid.org/0000-0001-9787-3948

Dyble M

Burin G

Lukas D https://orcid.org/0000-0002-7141-3545

Lonsdorf E

Peleg O https://orcid.org/0000-0001-9481-7967

Watts H https://orcid.org/0000-0002-9888-825X

Weber J

Crump A https://orcid.org/0000-0003-4485-5740

Wallace I

Blankers T https://orcid.org/0000-0002-1893-8537

Pomiankowski A https://orcid.org/0000-0002-5171-8755

Rivoire O https://orcid.org/0000-0002-1820-1324

Biedermann P https://orcid.org/0000-0003-4234-5659

Haaland T https://orcid.org/0000-0002-6968-4514

Hopkins G http://orcid.org/0000-0003-1427-5315

Milne M

Santos S

Wei K https://orcid.org/0000-0002-1694-9582

Bonier F https://orcid.org/0000-0001-7245-0244

Frere C https://orcid.org/0000-0002-9671-2138

Farrell MJ

McGaughran A

Baird R

Stepney S https://orcid.org/0000-0003-3146-5401

Getahun M https://orcid.org/0000-0001-5902-9236

Yong L

Gustison M

Palaoro A https://orcid.org/0000-0002-8629-0728

Scofield R https://orcid.org/0000-0002-7510-6980

Koop JAH

Burnett N https://orcid.org/0000-0003-4438-8062

Cade D https://orcid.org/0000-0003-3641-1242

Marsh J

Allen J https://orcid.org/0000-0002-0950-0429

Thomson J

Filipiak M https://orcid.org/0000-0001-7438-4885

Wells D

Stevenson T https://orcid.org/0000-0003-2644-9685

Ramesh V

Elemans C

Lambert M https://orcid.org/0000-0003-2318-6445

Clark N https://orcid.org/0000-0001-7131-3301

Satoh N https://orcid.org/0000-0002-4480-3572

Savage A

Matsuzawa T https://orcid.org/0000-0002-8147-2725

Sundberg L-R http://orcid.org/0000-0003-3510-4398

Blankers T https://orcid.org/0000-0002-1893-8537

Feit B https://orcid.org/0000-0002-1579-7695

Goymann W https://orcid.org/0000-0002-7553-5910

Tüzün N

Derex M https://orcid.org/0000-0002-1512-6496

Stoffer B

Fisher K

Smith J

Aichelman H https://orcid.org/0000-0001-6999-5518

Rummel A https://orcid.org/0000-0003-2627-8038

McCullogh I

Rushworth C

Améndola L

Alagador D

Pérez-Asensio JN https://orcid.org/0000-0002-1393-7412

Knowles SCL

Chenoweth S https://orcid.org/0000-0002-8303-9159

Salmón P https://orcid.org/0000-0001-9718-6611

Branch H

Reimer J https://orcid.org/0000-0003-0453-8804

Nokelainen O

Katzir G

Jagielska N https://orcid.org/0000-0001-7602-5878

Theunissen F

Armstrong JB

Neate-Clegg M https://orcid.org/0000-0001-9753-6765

Huang Y

Bouchet P

Dias M

Craven D https://orcid.org/0000-0003-3940-833X

Lehmann L https://orcid.org/0000-0001-9549-9577

Hughes M

Hellmann J https://orcid.org/0000-0003-0479-3960

Anderson S https://orcid.org/0000-0003-3096-1120

Lamont T

Seshadri A https://orcid.org/0000-0001-6128-3751

Morlo M

Harrison E https://orcid.org/0000-0002-2050-4631

Tomášek O https://orcid.org/0000-0002-2657-5916

Fogg L https://orcid.org/0000-0002-5378-8781

Kraay A https://orcid.org/0000-0002-7042-7048

Upchurch P

Debarre F https://orcid.org/0000-0003-2497-833X

Kortessis N

Juntti SA

Mullon C https://orcid.org/0000-0002-9875-4227

Barnett J https://orcid.org/0000-0001-9789-4132

Singhal S

Holwell G https://orcid.org/0000-0002-6059-6032

Phillips RD

Schurger A

Negishi J

Grieshop K https://orcid.org/0000-0001-8925-5066

Schindler D

de Miranda J https://orcid.org/0000-0002-0335-0386

Forstmeier W https://orcid.org/0000-0002-5984-8925

Blumstein D https://orcid.org/0000-0001-5793-9244

Zeng L-Q

Kellermann J

Reid J https://orcid.org/0000-0002-5007-7343

Lahdenperä M

Camerlink I https://orcid.org/0000-0002-3427-2210

McAuley T

Podos J https://orcid.org/0000-0002-2546-7999

Vitousek M https://orcid.org/0000-0003-2855-0832

Dudash M

Smith R

Sugiyama T

Hall J https://orcid.org/0000-0002-4896-4592

Dukas R https://orcid.org/0000-0003-4533-8542

Kulahci I https://orcid.org/0000-0003-0104-0365

Hemmings N https://orcid.org/0000-0003-2418-3625

Raguso R https://orcid.org/0000-0003-2066-3269

Price T https://orcid.org/0000-0002-4423-8999

Harpur BA

Barraquand F https://orcid.org/0000-0002-4759-0269

Sadovsky Y

Campbell P https://orcid.org/0000-0001-7660-9814

Parodi F

Kanwisher N https://orcid.org/0000-0003-3853-7885

Patten M

Waters J https://orcid.org/0000-0002-1514-7916

Kilpatrick A https://orcid.org/0000-0002-3612-5775

Rozen-Rechels D

Nilsson J-Å https://orcid.org/0000-0001-8982-1064

Heleno R

Smith M https://orcid.org/0000-0001-5660-1727

Brand C https://orcid.org/0000-0003-1285-2174

O'Connor M https://orcid.org/0000-0001-9583-1592

Griffith S https://orcid.org/0000-0001-7612-4999

Matheus J

McQueen A

Garlovsky M https://orcid.org/0000-0002-3426-4341

Rößler D https://orcid.org/0000-0003-4678-2231

Pujol B

Malik A

Keiser C https://orcid.org/0000-0002-4936-7810

Grunst M https://orcid.org/0000-0002-3425-4020

Wright D https://orcid.org/0000-0003-2329-2635

Alves-dos-Santos I

Colston T https://orcid.org/0000-0002-1127-1207

Breed G

Pereira Lopes R

Warren P

Eastwood J https://orcid.org/0000-0002-5294-3321

Clay T

Walker A

Marino C https://orcid.org/0000-0003-0470-6062

Bauchinger U

Kroumi D https://orcid.org/0000-0003-4861-0361

Kotler B

Miyanishi H

Wilts B https://orcid.org/0000-0002-2727-7128

Rice A https://orcid.org/0000-0002-5475-8226

Villa S https://orcid.org/0000-0001-5097-6026

Fiocca K https://orcid.org/0000-0002-5708-0641

Aguilera F https://orcid.org/0000-0003-3235-931X

Ottoni E https://orcid.org/0000-0003-1288-8345

Oppel S

Brown L https://orcid.org/0000-0003-3647-5879

Momigliano P https://orcid.org/0000-0001-6390-6094

Telfer S

Chari LD

Campbell C

Haaland T

Duboscq J http://orcid.org/0000-0002-6342-6709

Saleh F https://orcid.org/0000-0002-9165-985X

McGuigan D

Burness G

Bowers E https://orcid.org/0000-0003-4488-1463

Das B https://orcid.org/0000-0003-0855-262X

Schmid L

Bicknell RDC

Bodawatta K https://orcid.org/0000-0002-6095-9059

Roberts R

van der Marel A https://orcid.org/0000-0003-3942-8314

Owens G https://orcid.org/0000-0002-4019-5215

Strang C

Trinitapoli J

Howard M

Tucker M

Peer B https://orcid.org/0000-0002-4423-0508

Wainwright P

Brill R

Zattara E https://orcid.org/0000-0002-9947-9036

Rubenstein D https://orcid.org/0000-0002-4999-3723

Alizon S https://orcid.org/0000-0002-0779-9543

McHenry M https://orcid.org/0000-0001-5834-674X

Festa-Bianchet M

Mann J

Reynolds A https://orcid.org/0000-0002-4081-2754

Ramasindrazana B

Kendall W

Foo YZ https://orcid.org/0000-0001-7627-2991

Parslew B https://orcid.org/0000-0002-0070-488X

Detrain C

Tort L

Stearns S

Orlova M https://orcid.org/0000-0001-9920-8730

Picciulin M

Vasey P

Lennon J https://orcid.org/0000-0003-3126-6111

Laaksonen T

Quigley C https://orcid.org/0000-0002-7522-4426

Donahue M https://orcid.org/0000-0002-7529-001X

Soreq H https://orcid.org/0000-0002-0955-526X

Winkler L https://orcid.org/0000-0003-3597-6540

Somerville I

Vinebrooke R

Thomson J

Bernard J

Gearty W https://orcid.org/0000-0003-0076-3262

Laskowski K https://orcid.org/0000-0003-1523-9340

Gillingham M https://orcid.org/0000-0002-7935-9539

Narbona E

Holt R https://orcid.org/0000-0002-6685-547X

Quist CV

Liberti J

Langergraber K

Tarpy D

Kohler I

Mullon C https://orcid.org/0000-0002-9875-4227

Naya D https://orcid.org/0000-0002-8311-9263

Parsch J

Careau V https://orcid.org/0000-0002-2826-7837

Todgham A

Festa-Bianchet M

Lambert O

Reddon A https://orcid.org/0000-0002-3193-0388

Clarkson E

Sakata J https://orcid.org/0000-0003-2285-9310

Perez-Tris J https://orcid.org/0000-0001-5535-3100

Chapuisat M https://orcid.org/0000-0001-7207-199X

Forsman J

Turner A

Brown C

Norris D https://orcid.org/0000-0003-4874-1425

Tsang LM https://orcid.org/0000-0002-5029-5690

Osváth G

Beheim B

Tello-Ramos M https://orcid.org/0000-0003-0945-3498

Berv J

Rodriguez Flores P https://orcid.org/0000-0003-1555-9598

Johnson T

Henry L https://orcid.org/0000-0002-5706-1328

Vindas M https://orcid.org/0000-0002-3996-0952

Wilson R https://orcid.org/0000-0003-3177-0107

Schenkel M https://orcid.org/0000-0002-7721-319X

Timóteo S https://orcid.org/0000-0003-2417-3259

Santos Matos G

Hill J

Hsu C

Marcondes R

Gronenberg W

McKinnon J

McClean D https://orcid.org/0000-0002-5857-9081

ten Cate C https://orcid.org/0000-0002-4021-8915

Lanzetti A https://orcid.org/0000-0003-1364-0544

Ram Y

Knauer A https://orcid.org/0000-0002-4869-4596

Wotton K

Grundel R

Bertram J

McLean D https://orcid.org/0000-0001-6229-7063

Delhey K https://orcid.org/0000-0001-5190-5406

Jenni-Eiermann S

Garlovsky M https://orcid.org/0000-0002-3426-4341

Krause T https://orcid.org/0000-0002-8327-3711

Postma E https://orcid.org/0000-0003-0856-1294

Longdon B https://orcid.org/0000-0001-6936-1697

Rubio De Casas R http://orcid.org/0000-0003-4276-4968

Raymond M

Gilburn A

Ritchie M

Stenum J

Clark C https://orcid.org/0000-0001-7943-9291

Islam S

Webb S

Harville E

Molloy EK https://orcid.org/0000-0001-5553-3312

Ushio M https://orcid.org/0000-0003-4831-7181

Caspers S

Ghedini G https://orcid.org/0000-0002-5156-2009

Olsson M https://orcid.org/0000-0002-4130-1323

Gómez P https://orcid.org/0000-0003-2830-4105

Burchardt LS https://orcid.org/0000-0002-9210-7934

Theofanopoulou C https://orcid.org/0000-0003-2014-7563

Fink J

Moura R https://orcid.org/0000-0002-7911-4734

Murray-Stoker D

Stojanova B

Billionnet A

Mulhair P https://orcid.org/0000-0003-3311-4883

Burin G

Provini P

Ferdy J-B

Weiss A

Conti-Jerpe IE

Nishitani S https://orcid.org/0000-0002-6234-295X

Lukhtanov V https://orcid.org/0000-0003-2856-2075

Stefanescu C https://orcid.org/0000-0001-8952-7869

Williams R

Sadovsky Y

Satake A https://orcid.org/0000-0002-0831-8617

Shizuka D https://orcid.org/0000-0002-0478-6309

Mable B

Arikawa K https://orcid.org/0000-0002-4365-0762

Fieder M https://orcid.org/0000-0002-3257-8902

Condamine F https://orcid.org/0000-0003-1673-9910

Fu F

Streinzer M

Oda G https://orcid.org/0000-0001-7776-5368

O'Hara T https://orcid.org/0000-0003-0885-6578

Dunlop R https://orcid.org/0000-0002-0427-6317

Baird E https://orcid.org/0000-0003-3625-3897

Brand J https://orcid.org/0000-0003-3312-941X

Van Wassenbergh S https://orcid.org/0000-0001-5746-4621

Yi Y https://orcid.org/0000-0003-0845-5635

Codd J https://orcid.org/0000-0003-0211-1786

Breuer T

Gonzalez-Tokman D https://orcid.org/0000-0001-7251-5773

Fay R https://orcid.org/0000-0002-7202-367X

Scow K https://orcid.org/0000-0002-2649-1122

Elowe C https://orcid.org/0000-0002-0983-6352

VanRoy P

Cheng L

Pincheira-Donoso D

Frank S https://orcid.org/0000-0001-7348-7794

Moore T https://orcid.org/0000-0003-0867-4512

Emery Thompson M https://orcid.org/0000-0003-2451-6397

Robinson M

Nielsen M

Allen B https://orcid.org/0000-0003-0282-6407

Geffroy B

Fearon M https://orcid.org/0000-0001-6849-4834

carlen e

Allan E

Rubolini D

Gehman A-L https://orcid.org/0000-0003-0595-9950

Cronin T https://orcid.org/0000-0001-7375-9382

Bengtsson-Palme J

Kellermann V https://orcid.org/0000-0002-9859-9642

Elvidge C

Somanathan H https://orcid.org/0000-0002-4803-3894

Brockington S

De Hevia MD https://orcid.org/0000-0002-1495-3721

McGuire J https://orcid.org/0000-0003-0739-309X

Barve S https://orcid.org/0000-0001-5840-8023

Mauvisseau Q

Rebollar EA

van de Meij S

Cai C https://orcid.org/0000-0002-9283-8323

Noguera J

McDonald G

Wilfert L https://orcid.org/0000-0002-6075-458X

Simon R

Reiskind M

Johnson S https://orcid.org/0000-0002-6294-8302

Antia R

Levy O

Mellin C https://orcid.org/0000-0002-7369-2349

Lunau K https://orcid.org/0000-0001-5184-4201

Fricke C https://orcid.org/0000-0002-0691-6779

Herrera J https://orcid.org/0000-0002-0633-0575

Smith J https://orcid.org/0000-0002-3342-4454

Brock C

Becker D https://orcid.org/0000-0003-4315-8628

Osada N https://orcid.org/0000-0003-0180-5372

Zapalski M https://orcid.org/0000-0002-9853-3707

Sunagawa G https://orcid.org/0000-0002-3952-2143

Dick C

Zipple M

Toxopeus J

Ouyang J

Shaw R

Cicchini GM https://orcid.org/0000-0002-3303-0420

Oestreich W

Lambers H

Horita Y https://orcid.org/0000-0002-9864-9988

Labonte D https://orcid.org/0000-0002-1952-8732

Owen-Smith N

Sollmann R

Getahun M https://orcid.org/0000-0001-5902-9236

Bujan J https://orcid.org/0000-0002-7938-0266

Quesada-Moraga E

Caillaud D

Mundry R

Carlen E

Faillace C

Wikberg E

Hillyer J https://orcid.org/0000-0002-3178-0201

Gershman S https://orcid.org/0000-0003-4451-820X

Reindl E

Baca M https://orcid.org/0000-0003-2174-3914

Palmer-Young E https://orcid.org/0000-0002-9258-2073

Lane S https://orcid.org/0000-0002-3797-3178

Mutshinda CM https://orcid.org/0000-0001-9671-7812

Rovenolt F https://orcid.org/0000-0001-5877-0460

Vargas S

Potvin D https://orcid.org/0000-0001-9296-9297

Uszko W

Dyer A https://orcid.org/0000-0002-2632-9061

Bohm S https://orcid.org/0000-0002-5720-3672

Sanghvi K

Van Buren T

Schachner E

Boyer L https://orcid.org/0000-0002-6976-1305

Cáceres N

Sasaki M https://orcid.org/0000-0001-5560-5363

Rodríguez R https://orcid.org/0000-0003-0262-0839

Chhetri D

de Boer R

Rawlence N https://orcid.org/0000-0001-6968-6643

O'Shea RP https://orcid.org/0000-0003-3132-7199

Ayala Lara R https://orcid.org/0000-0002-3239-0854

Fang F

Milne M

Peer B https://orcid.org/0000-0002-4423-0508

Schnaitmann C

Garroway C https://orcid.org/0000-0002-0955-0688

Smaldino P https://orcid.org/0000-0002-7133-5620

Burns R

Vidal-Dupiol J

Boomsma PJ

He P https://orcid.org/0000-0002-7176-701X

Golbuu Y

Schmidt M

Wielgus E

Wortel M https://orcid.org/0000-0002-3191-2710

Schweinfurth M https://orcid.org/0000-0003-2066-7892

Gardner A https://orcid.org/0000-0002-1304-3734

Lomáscolo S https://orcid.org/0000-0002-4422-7851

Wei N https://orcid.org/0000-0002-7345-501X

Alaasam V https://orcid.org/0000-0003-1670-6780

Gray P

Peterman S https://orcid.org/0000-0002-6498-7938

Scordato E https://orcid.org/0000-0003-0224-8280

Miller A

Hartfield M

Stieglitz J https://orcid.org/0000-0001-5985-9643

Rand T

Morita M https://orcid.org/0000-0002-0170-1226

Herrel A

Mallinger R

Kimmitt A https://orcid.org/0000-0003-0044-8297

Yeaman S

Jacobsen M

Truchy A https://orcid.org/0000-0002-2423-8924

Deme G

Waters C

Xu X

Bjørge A

Caves E https://orcid.org/0000-0003-3497-5925

Meselson M

Hunt G https://orcid.org/0000-0001-6430-5020

Peleg O https://orcid.org/0000-0001-9481-7967

Albrecht J https://orcid.org/0000-0002-9708-9413

Schausberger P https://orcid.org/0000-0002-1529-3198

Jackson J

Remeš V

Grzędzicka E https://orcid.org/0000-0002-3954-4895

Hall J https://orcid.org/0000-0002-4896-4592

Zanders SE

Ellner S https://orcid.org/0000-0002-8351-9734

Poblete J

Vining A

Pyenson N https://orcid.org/0000-0003-4678-5782

Levin M https://orcid.org/0000-0001-7292-8084

Parker E

Flammang B https://orcid.org/0000-0003-0049-965X

Campbell P https://orcid.org/0000-0001-7660-9814

Cory J https://orcid.org/0000-0002-4637-6439

Thorogood R https://orcid.org/0000-0001-5010-2177

Ruff C https://orcid.org/0000-0002-2932-3634

El-Gabbas A

Wilkinson G https://orcid.org/0000-0001-7799-8444

Silverman A

Filippi P https://orcid.org/0000-0002-2990-9344

Krueger-Hadfield S

Ida T

Pellis S

Elston D

De Lisle S https://orcid.org/0000-0001-9587-8665

Hübler N https://orcid.org/0000-0002-0013-563X

Tautz D

Madimenos FC

Johnson L

Klassen J

Cockburn A

Farmer C https://orcid.org/0000-0003-4532-7755

Weithoff G

Senner N https://orcid.org/0000-0003-2236-2697

Larson S

Matthews B

Minamoto T

Fresneau N https://orcid.org/0000-0003-3427-6032

Bitter M https://orcid.org/0000-0001-7607-2375

Salahshour M https://orcid.org/0000-0001-5354-9839

Whitehouse J https://orcid.org/0000-0003-2607-5492

Schultheiss P https://orcid.org/0000-0002-6906-3507

Valente L https://orcid.org/0000-0003-4247-8785

Evans S

Devaud J-M https://orcid.org/0000-0002-9873-2204

Okamoto D https://orcid.org/0000-0001-8988-4567

Sargent R https://orcid.org/0000-0001-8736-5712

Price T https://orcid.org/0000-0002-4423-8999

MacNulty D

Mancini P

Cronin A https://orcid.org/0000-0003-2075-1138

Hongoh Y

Muralidhar P

Ridley A https://orcid.org/0000-0001-5886-0992

van der Hoek Y https://orcid.org/0000-0003-3979-2896

Blanckenhorn W https://orcid.org/0000-0002-0713-3944

Rosario M https://orcid.org/0000-0001-9969-6746

Boisseau R

Lopez-Uribe MM https://orcid.org/0000-0002-8185-2904

Currie S https://orcid.org/0000-0001-5956-0481

Dougherty L https://orcid.org/0000-0003-1406-0680

Rodrigues M

Webb B https://orcid.org/0000-0002-8336-6926

Uwe M

Whittington E

Manning J https://orcid.org/0000-0001-8130-5460

Forrest J

O'Neal ME

Perlman S

Akcay C https://orcid.org/0000-0003-0635-9586

Rouyer F

Ålund M https://orcid.org/0000-0003-2861-9721

Slotnick B

Robinson G

‪Kapli ‪P

Seebacher F https://orcid.org/0000-0002-2281-9311

Triki Z

Cohen S

Bessho K https://orcid.org/0000-0002-5911-6855

Gérard M https://orcid.org/0000-0002-2485-0662

Tharreau D

Dhawale A

Moazen M https://orcid.org/0000-0002-9951-2975

Purnell M

Buatois L https://orcid.org/0000-0001-9523-750X

Kappel S

Farnsworth A

Tarwater C

Campos F https://orcid.org/0000-0001-9826-751X

Barneche D

Shuai Z

Tompros A

Dunhill A https://orcid.org/0000-0002-8680-9163

Durland E https://orcid.org/0000-0003-1322-6810

Baldwin M

Keller R

Borensztein M https://orcid.org/0000-0002-4378-5018

Brandl S

Prugh L

Harville E

Sukhum K https://orcid.org/0000-0002-4428-5381

Eguchi T

Tong C https://orcid.org/0000-0001-5202-5507

Uehling J

Sohn H https://orcid.org/0000-0001-8593-7473

Luo M https://orcid.org/0000-0002-6340-7786

Donoghue P https://orcid.org/0000-0003-3116-7463

Ellis S https://orcid.org/0000-0001-9019-6040

Nilsson L

Cheptou P-O https://orcid.org/0000-0002-5739-5176

Cooper R https://orcid.org/0000-0003-0172-4708

Erickson P https://orcid.org/0000-0001-8420-995X

Graham C

Jones O

Wilber M

Metzler D

Zanoli A https://orcid.org/0000-0002-9402-5617

Santicchia F https://orcid.org/0000-0003-4814-9632

Bruijn S https://orcid.org/0000-0003-0290-2131

Thoré E

Metzger B

Zinsstag J

Aguirre JD

Pitulko VV

Marko P

Maestripieri D

Suggitt A

Manser M

Weir J

García-Rodríguez A https://orcid.org/0000-0002-9831-2963

McHenry M https://orcid.org/0000-0001-5834-674X

Dunn D

Vorobyev M https://orcid.org/0000-0001-7615-5816

Nishitani S https://orcid.org/0000-0002-6234-295X

Srinivasan U https://orcid.org/0000-0002-8040-1124

Alto B https://orcid.org/0000-0001-7054-2081

Stayton T

Weber J

Verhulst S https://orcid.org/0000-0002-1143-6868

Barclay K

Martin T

Stahl A

De Hevia MD https://orcid.org/0000-0002-1495-3721

Santos K

Szabo B https://orcid.org/0000-0002-3226-8621

Nabholz B

Gloag R https://orcid.org/0000-0002-2037-4267

Dreyer D https://orcid.org/0000-0003-4344-8596

López-Estrada K

Anderson JP

De Dreu C https://orcid.org/0000-0003-3692-4611

Lukas D https://orcid.org/0000-0002-7141-3545

Boudry P

Pagán I https://orcid.org/0000-0001-8876-1194

Nelson-Flower M https://orcid.org/0000-0001-6753-9252

Sugiyama T

Pain R https://orcid.org/0000-0002-5354-6301

Knief U https://orcid.org/0000-0001-6959-3033

Collar D

Organ J

Henrique D

Moran R https://orcid.org/0000-0001-6831-4530

Witt C https://orcid.org/0000-0003-2781-1543

Wallace I

Fonio E

Wiens J https://orcid.org/0000-0003-4243-1127

Conklin J

Escobar LE https://orcid.org/0000-0001-5735-2750

Ghalambor C

Amodio P

McCabe B

Kutzer M https://orcid.org/0000-0002-8696-6978

Van Iten H

Dey C https://orcid.org/0000-0003-4947-8972

McConkey K

Ulrich Y https://orcid.org/0000-0001-9380-7863

Cisek P

Thuenken T https://orcid.org/0000-0002-6518-8687

Kantsa A

Weiss A

D'aniello E

Gray D https://orcid.org/0000-0002-4439-561X

Ashander J https://orcid.org/0000-0002-1841-4768

Khattar G

Ruggera R

Martínez-Núñez C https://orcid.org/0000-0001-7814-4985

Mair RG

Gershman S https://orcid.org/0000-0003-4451-820X

Bahram M

Hoelzel R https://orcid.org/0000-0002-7265-4180

Zhang J https://orcid.org/0000-0001-6141-1290

Parker DJ

Siracusa ER https://orcid.org/0000-0003-4205-7278

Mann J

Elliott K

Rosenberg K

Falk J

Homberg U

Perc M https://orcid.org/0000-0002-3087-541X

Fiedler K

Allgeier J https://orcid.org/0000-0002-9005-6432

Manafzadeh A https://orcid.org/0000-0001-5388-7942

Derex M https://orcid.org/0000-0002-1512-6496

Leimar O https://orcid.org/0000-0001-8621-6977

Zuleta D

Brusch G https://orcid.org/0000-0001-7740-6066

Villanueva R

Yorzinski J https://orcid.org/0000-0002-4193-6695

Dargent F https://orcid.org/0000-0002-4510-0086

Kench P

Conaco C

Lehmann L https://orcid.org/0000-0001-9549-9577

Swan C

Zapalski M https://orcid.org/0000-0002-9853-3707

Shogren E https://orcid.org/0000-0002-8619-3549

Dhungana A

Kibbe M

Templeton C https://orcid.org/0000-0001-8701-3186

Song C https://orcid.org/0000-0001-7490-8626

Guo K https://orcid.org/0000-0001-6765-1957

Burness G

Chittaro P

Gutiérrez-Cánovas C

Orben R https://orcid.org/0000-0002-0802-407X

Huang S-Q

Huisman J https://orcid.org/0000-0002-1782-8109

Berbel-Filho W https://orcid.org/0000-0001-6991-4685

Dudley R https://orcid.org/0000-0003-3707-5682

Seymoure B https://orcid.org/0000-0003-3596-1832

Grossnickle DM

McCurry M https://orcid.org/0000-0002-2452-1404

Berec L https://orcid.org/0000-0002-2419-3324

Spaak J https://orcid.org/0000-0001-5157-9188

Sattenspiel L https://orcid.org/0000-0003-4534-3392

Hawley D https://orcid.org/0000-0001-9573-2914

Wolff J

Thorogood R https://orcid.org/0000-0001-5010-2177

Wood B https://orcid.org/0000-0002-8187-9429

Britton D https://orcid.org/0000-0002-9029-7527

Clemente C https://orcid.org/0000-0001-8174-3890

Maldonado K https://orcid.org/0000-0001-8946-7560

Santema P https://orcid.org/0000-0002-5765-3067

McDaniel S https://orcid.org/0000-0002-5435-7377

Ivimey-Cook E https://orcid.org/0000-0003-4910-0443

Espinosa J https://orcid.org/0000-0003-0780-2762

Bardin J https://orcid.org/0000-0003-2382-4259

Turner L

Cueva del Castillo R https://orcid.org/0000-0002-6385-0729

Pearse I https://orcid.org/0000-0001-7098-0495

Rodríguez R https://orcid.org/0000-0003-0262-0839

Matos M

Klimesova J

Keays D

Courbin N http://orcid.org/0000-0002-6428-5012

Horita Y https://orcid.org/0000-0002-9864-9988

Reddon A https://orcid.org/0000-0002-3193-0388

Schmid D

Nöbel S https://orcid.org/0000-0002-1850-8895

Pulgar J

Thompson González N

Krishnan A https://orcid.org/0000-0002-8317-4991

Müller J

Farris D https://orcid.org/0000-0002-6720-1961

Whiten A https://orcid.org/0000-0003-2426-5890

Zipple M

Giffin K

Kristensen T

Simpson C https://orcid.org/0000-0003-0719-4437

Walters E https://orcid.org/0000-0002-9414-5758

Myers J

Doublet V

Luo M https://orcid.org/0000-0002-2975-5218

Veliz D

Butterfield N https://orcid.org/0000-0002-3046-7520

Tomášek O https://orcid.org/0000-0002-2657-5916

Macartney E https://orcid.org/0000-0003-3866-143X

Haaland T

Hawley D https://orcid.org/0000-0001-9573-2914

Stroud J https://orcid.org/0000-0003-0734-6795

Mello C

Cheviron Z

Tanaka M https://orcid.org/0000-0003-2198-1402

Cox R

Herranz M

Heleno R

Heinicke M https://orcid.org/0000-0002-6021-8058

Liu B

Theofanopoulou C https://orcid.org/0000-0003-2014-7563

Barabas G

Bernier P-M

Danforth B

Janisch J https://orcid.org/0000-0003-3300-6292

Robertson A

Vasudeva R https://orcid.org/0000-0002-3831-0384

Caro T https://orcid.org/0000-0001-6804-8519

Zeng H https://orcid.org/0000-0002-5728-7896

Garzke J https://orcid.org/0000-0002-5881-1813

Fouke B https://orcid.org/0000-0001-7698-7241

Cahill AE

Toll K

Chipman A https://orcid.org/0000-0001-6696-840X

Willis K

Martin-Duran J https://orcid.org/0000-0002-2572-1061

Roberts G https://orcid.org/0000-0001-5954-3243

McHenry M https://orcid.org/0000-0001-5834-674X

Farji-Brener A https://orcid.org/0000-0001-7251-3866

Dreissig S https://orcid.org/0000-0002-4766-9698

Haegeman B

Alvarez Perez JA

van der Marel A https://orcid.org/0000-0003-3942-8314

Careau V https://orcid.org/0000-0002-2826-7837

Cutter A https://orcid.org/0000-0001-7141-0013

Lane S

Dediu D https://orcid.org/0000-0002-0704-6365

Récapet C https://orcid.org/0000-0001-5414-8412

Bruzos AL

Kirschel A https://orcid.org/0000-0003-4379-7956

Ridley A https://orcid.org/0000-0001-5886-0992

Szathmary E https://orcid.org/0000-0001-5227-2997

Ushio M https://orcid.org/0000-0003-4831-7181

Dunhill A https://orcid.org/0000-0002-8680-9163

Holmes J https://orcid.org/0000-0001-8804-2149

DeRuiter S

Maggioni D https://orcid.org/0000-0003-0508-3987

Thorley J https://orcid.org/0000-0002-8426-610X

Fowler-Finn K https://orcid.org/0000-0003-2951-3283

Reid B https://orcid.org/0000-0003-4063-3956

Peng F https://orcid.org/0000-0002-1637-5611

Talavera G https://orcid.org/0000-0003-1112-1345

Cavigelli S https://orcid.org/0000-0002-6344-9759

Liang W https://orcid.org/0000-0002-0004-9707

Aich U https://orcid.org/0000-0003-2576-0922

Daly J https://orcid.org/0000-0003-2052-4672

Narendra A https://orcid.org/0000-0002-1286-5373

Korb J https://orcid.org/0000-0001-9577-9376

Lev Ari S

Parker B

Döring T https://orcid.org/0000-0001-9074-5978

Cressler C https://orcid.org/0000-0002-6281-2798

Fox A https://orcid.org/0000-0002-7852-4539

Hung K-L https://orcid.org/0000-0002-1557-3958

Riva F https://orcid.org/0000-0002-1724-4293

Jackson J

Tyack P https://orcid.org/0000-0002-8409-4790

Koprivnikar J https://orcid.org/0000-0001-8410-1041

Pespeni M https://orcid.org/0000-0001-5447-6678

Dukas R https://orcid.org/0000-0003-4533-8542

Huckstadt L https://orcid.org/0000-0002-2453-7350

Youngsteadt E https://orcid.org/0000-0003-2032-9674

Campbell B

Weller H

Pratt RB

Servedio M

Paracchini S https://orcid.org/0000-0001-9934-8602

Dujon A https://orcid.org/0000-0002-1579-9156

Esteve J https://orcid.org/0000-0003-2806-2695

Didham R https://orcid.org/0000-0001-6685-7005

Powell M

Barve S https://orcid.org/0000-0001-5840-8023

Symons C https://orcid.org/0000-0003-4120-0327

Rainbow M

Henshaw J https://orcid.org/0000-0001-7306-170X

Hitchcock T https://orcid.org/0000-0002-6378-5023

Gaillard J-M

Iwanicki T https://orcid.org/0000-0003-1128-6203

Rouleau C

Thorogood R https://orcid.org/0000-0001-5010-2177

D'Amelio P https://orcid.org/0000-0002-4095-6088

Singer M

Vargas G

Olson ME

Tejero-Cicuéndez H https://orcid.org/0000-0001-8151-465X

Tyrrell L

Fiocca K https://orcid.org/0000-0002-5708-0641

Kingston A

Burdfield-Steel E

Harrison J https://orcid.org/0000-0001-5223-216X

Wybouw N https://orcid.org/0000-0001-7874-9765

Wotton K

Kuijper B https://orcid.org/0000-0002-7263-2846

Roth A https://orcid.org/0000-0002-7196-0186

Ligocki I

Stenum J

Harnik P https://orcid.org/0000-0001-6931-9931

Fu F

Dusek A https://orcid.org/0000-0001-8552-4089

Kaitala V https://orcid.org/0000-0001-5626-9752

Berner D

Clemens A https://orcid.org/0000-0001-5488-0902

Janda M

Peterman S https://orcid.org/0000-0002-6498-7938

Pomiankowski A https://orcid.org/0000-0002-5171-8755

Cummings M https://orcid.org/0000-0002-4873-5378

Moore T https://orcid.org/0000-0003-0867-4512

Marshall J

Hejnol A

Iossa G https://orcid.org/0000-0001-6813-4361

Montoya B https://orcid.org/0000-0001-5552-9831

Foulkes N

Härer A https://orcid.org/0000-0003-2894-5041

Białas J

Kulahci I https://orcid.org/0000-0003-0104-0365

Kulahci I https://orcid.org/0000-0003-0104-0365

Kelly D

Raupach M

Koch I

Bertolucci C https://orcid.org/0000-0003-0252-3107

Wilber M

Egan M

Smith D https://orcid.org/0000-0003-4367-6556

Chen C https://orcid.org/0000-0002-5035-4021

Harvey C https://orcid.org/0000-0002-2830-0844

Careau V https://orcid.org/0000-0002-2826-7837

Manohar S

Hughes B

Romero T

Rezende G https://orcid.org/0000-0002-8904-7598

Anderson JP

Green R https://orcid.org/0000-0001-8690-8914

Thompson J

Stothart M https://orcid.org/0000-0002-2863-908X

Abou Chakra M

Bojarska K

Fischer E https://orcid.org/0000-0002-2916-0900

Carere C

Shaw R

Lynch J

Lehtonen J https://orcid.org/0000-0001-5260-1041

Donelson J

Hu J https://orcid.org/0000-0003-1857-8700

Nilsson A https://orcid.org/0000-0002-3541-9835

Weber de Melo V https://orcid.org/0000-0001-9558-4042

Brehm A

Mangiamele LA https://orcid.org/0000-0003-4488-4904

Ma W-J https://orcid.org/0000-0003-2585-6406

Duarte R https://orcid.org/0000-0001-7059-3129

Schradin C https://orcid.org/0000-0002-2706-2960

Valente D https://orcid.org/0000-0001-6086-5135

Agrawal A https://orcid.org/0000-0003-0095-1220

Perc M https://orcid.org/0000-0002-3087-541X

Ebensperger L

Willems F

Gvozdik L

Regan C https://orcid.org/0000-0002-5458-5687

Kershenbaum A

Mulhair P https://orcid.org/0000-0003-3311-4883

Briffa M https://orcid.org/0000-0003-2520-0538

Bindler R

Turner J https://orcid.org/0000-0001-5449-5449

Magalhães S

Ruhi A

Mazzocchi MG

Yang Y

Selbach C https://orcid.org/0000-0002-7777-6515

Marshall A

Sastri A

Rubio De Casas R http://orcid.org/0000-0003-4276-4968

Roney J https://orcid.org/0000-0002-5389-383X

Santos K

Mundry R

Kaiser D https://orcid.org/0000-0002-9007-3160

Carey D

Wood S https://orcid.org/0000-0002-8273-4045

Muscarella R

Cooney D https://orcid.org/0000-0001-8643-0893

Bernatchez L

Galván I https://orcid.org/0000-0002-6523-8592

Smith N

Gosler A

Dupuis J

Amaro F https://orcid.org/0000-0002-0061-477X

Waldvogel A-M

Pintar M https://orcid.org/0000-0003-0165-3882

Zeng Q

Burns R

Pannell J https://orcid.org/0000-0002-0098-7074

Harrison R

Timóteo S https://orcid.org/0000-0003-2417-3259

Papeo L

Title P

Kelly L https://orcid.org/0000-0002-9736-0517

Aerts P https://orcid.org/0000-0002-6867-5421

Doublet V

Narbona E

Pereira Lopes R

Brietbart S

Wilson A

Venkadesan M https://orcid.org/0000-0001-5754-7478

Zhou X https://orcid.org/0000-0002-2385-8224

Cooney C https://orcid.org/0000-0002-4872-9146

ten Cate C https://orcid.org/0000-0002-4021-8915

Haaland T https://orcid.org/0000-0002-6968-4514

Bretman A https://orcid.org/0000-0002-4421-3337

Fieder M https://orcid.org/0000-0002-3257-8902

Hector T

Murray G

Pazhenkova E

Amador-Vargas S https://orcid.org/0000-0001-8738-4876

Sun S-Jyun https://orcid.org/0000-0002-7859-9346

Kojima D

Bousquet C https://orcid.org/0000-0003-1650-8004

Aichelman H https://orcid.org/0000-0001-6999-5518

Rubin G

Anaya-Rojas J https://orcid.org/0000-0002-0688-3401

Ulrich W

Fior S

Richards C https://orcid.org/0000-0001-7848-5165

Ricci B https://orcid.org/0000-0002-8661-4200

Hall J https://orcid.org/0000-0002-4896-4592

Larissa Boesing A

Coltman D

Broderick N https://orcid.org/0000-0002-6830-9456

Deme G

Aniceto S https://orcid.org/0000-0001-5101-9375

Rohner N

Jungwirth A

Bleasdale B

Rovenolt F https://orcid.org/0000-0001-5877-0460

Buck C

Shultz S https://orcid.org/0000-0002-7135-4880

Shabangu FW https://orcid.org/0000-0002-3668-3566

Liao C-C https://orcid.org/0000-0002-0803-0234

DuVal E

Hughes A https://orcid.org/0000-0003-2677-1965

Freeberg T https://orcid.org/0000-0003-3807-4147

Ecke F

Dong G

Balakrishnan R

Ganel T

Lanzetti A https://orcid.org/0000-0003-1364-0544

Limmathurotsakul D https://orcid.org/0000-0001-7240-5320

Hedenström A https://orcid.org/0000-0002-1757-0945

Michelangeli M

Prado A https://orcid.org/0000-0003-2905-4769

Czarnecka M

Todd P

Poertner H

Holeski L

Chippindale AK

Hsieh C https://orcid.org/0000-0002-3715-4113

Johannesen A

Van Opzeeland I

Mortimer B

Burton G https://orcid.org/0000-0001-8677-4143

Samant M

Gaigher R

Talbot C https://orcid.org/0000-0003-4604-6891

Owens H https://orcid.org/0000-0003-0071-1745

Forstmeier W https://orcid.org/0000-0002-5984-8925

Josic K

Menzel F https://orcid.org/0000-0002-9673-3668

Marcinkowska U

Merondun J

Kellermann V https://orcid.org/0000-0002-9859-9642

Momigliano P https://orcid.org/0000-0001-6390-6094

Fresneau N https://orcid.org/0000-0003-3427-6032

Munshi-South J https://orcid.org/0000-0002-8067-4341

Gabor C https://orcid.org/0000-0001-7584-1451

Cheng W

Heffernan C

Metcalfe N https://orcid.org/0000-0002-1970-9349

Grogan K https://orcid.org/0000-0002-0112-1766

Lund A

Champredon D https://orcid.org/0000-0002-7090-8757

Oosthuizen C

Cortés-Ortiz L

Hawley D https://orcid.org/0000-0001-9573-2914

Wang R https://orcid.org/0000-0003-4652-2149

Volodina E https://orcid.org/0000-0001-9755-4576

Spoelstra K https://orcid.org/0000-0001-8614-4387

Laborda P

Liénard MA https://orcid.org/0000-0003-3193-3666

Barbosa M https://orcid.org/0000-0003-0327-9580

Segner H

Hamilton I https://orcid.org/0000-0002-1198-3431

Duffy M https://orcid.org/0000-0002-8142-0802

Rasher D

DeLong J https://orcid.org/0000-0003-0558-8213

Van Goor J https://orcid.org/0000-0001-7498-9315

Haig D

Herrera N

Pisanski K https://orcid.org/0000-0003-0992-2477

McDonald G

Seymour M

Fan P-F https://orcid.org/0000-0003-4747-5727

Ragsdale E https://orcid.org/0000-0002-9367-8552

Canal D

Pacheco A https://orcid.org/0000-0002-7899-9657

Charpentier C

Hass A

Pettersen A https://orcid.org/0000-0001-6191-6563

Hitchcock T https://orcid.org/0000-0002-6378-5023

Zhang Y

Akan O-D https://orcid.org/0000-0002-2758-1947

Shao Y

Dukas R https://orcid.org/0000-0003-4533-8542

Bonier F https://orcid.org/0000-0001-7245-0244

Bendrey R

Bonnet X https://orcid.org/0000-0001-6150-8199

Stiling P

Auerbuch V

Alves L https://orcid.org/0000-0002-8944-1851

Morita M https://orcid.org/0000-0002-0170-1226

Gleeson B https://orcid.org/0000-0001-6911-6202

Ord T https://orcid.org/0000-0002-2608-2150

Yukilevich R

Diniz-Filho JA https://orcid.org/0000-0002-0967-9684

Mateo J

Singer M

Barraquand F https://orcid.org/0000-0002-4759-0269

Webb B https://orcid.org/0000-0002-8336-6926

Schmitz O https://orcid.org/0000-0003-1515-2667

Ferrante M

Ferraguti M https://orcid.org/0000-0001-7481-4355

Downing P https://orcid.org/0000-0002-5286-3153

Liedvogel M https://orcid.org/0000-0002-8372-8560

Shuai Z

Martin-Duran J https://orcid.org/0000-0002-2572-1061

Fu F

Oestreich W

Beltran RS https://orcid.org/0000-0002-8520-1105

Rendueles O https://orcid.org/0000-0002-6648-1594

Thorogood R https://orcid.org/0000-0001-5010-2177

Katzir G

Schoerning E

Tallon-Baudry C

Hung K-L https://orcid.org/0000-0002-1557-3958

Elgar M https://orcid.org/0000-0003-0861-6064

Encinas-Viso F

Loued-Khenissi L https://orcid.org/0000-0003-3241-6492

Casagrande S https://orcid.org/0000-0002-4264-8062

Filipiak M https://orcid.org/0000-0001-7438-4885

Schenkel M https://orcid.org/0000-0002-7721-319X

Gibson A https://orcid.org/0000-0002-0867-4953

Wotton K

Cutts V https://orcid.org/0000-0002-4986-2934

Cahill AE

Nichols H https://orcid.org/0000-0002-4455-6065

Cannon S

Hawlena D

Myers C

de Angeli Dutra D https://orcid.org/0000-0003-2341-2035

Nash K

Girard F https://orcid.org/0000-0002-7846-2710

Molina Venegas R

Harvey E https://orcid.org/0000-0002-8601-7326

Maldonado-Chaparro A https://orcid.org/0000-0003-0853-4966

O'Hara T https://orcid.org/0000-0003-0885-6578

Girard-Buttoz C https://orcid.org/0000-0003-1742-4400

Samhita L

Boutin S

Hawley D https://orcid.org/0000-0001-9573-2914

Teder T https://orcid.org/0000-0001-6587-9325

Festa-Bianchet M

Simic G

Breed G

Nokelainen O

Wolff J

Baliga V https://orcid.org/0000-0002-9367-8974

Fricke E https://orcid.org/0000-0002-0520-4200

Grzędzicka E https://orcid.org/0000-0002-3954-4895

Svedberg J

Fuentes-Peñailillo F https://orcid.org/0000-0001-5658-7558

Petrullo L

Jordal B

Boynton P

Capilla-Lasheras P

Garcia Perez C

Ormerod S

Crespi B https://orcid.org/0000-0002-0362-392X

Clark C https://orcid.org/0000-0001-7943-9291

Foster-Dyer R https://orcid.org/0000-0001-9047-6851

Gallup Jr G

Maggioni D https://orcid.org/0000-0003-0508-3987

Waldman B https://orcid.org/0000-0003-0006-5333

Alderdice R

Hale R

Filippi P https://orcid.org/0000-0002-2990-9344

Sheard J

Mesoudi A https://orcid.org/0000-0002-7740-1625

Fuxjager M https://orcid.org/0000-0003-0591-6854

Teitelbaum C https://orcid.org/0000-0001-5646-3184

Leal CG https://orcid.org/0000-0002-0108-8572

Dawson W

Dahms H-U

Luebeck G

Bakasubramaniam K

Guralnick R

Dyer A https://orcid.org/0000-0002-2632-9061

Weckström J

Anderson J

Roast M https://orcid.org/0000-0002-9454-7562

O'Toole A

Garroway C https://orcid.org/0000-0002-0955-0688

Mann J

Fiocca K https://orcid.org/0000-0002-5708-0641

Bradler S

Garcia-RJ https://orcid.org/0000-0002-6665-9671

Theobald J https://orcid.org/0000-0001-7648-3772

Miller J

Daly J https://orcid.org/0000-0003-2052-4672

Gaillard J-M

Gebauer G

Lucon-Xiccato T https://orcid.org/0000-0002-1017-9230

Ahrens D https://orcid.org/0000-0003-3524-7153

Fowler J

Boddy A

Ramesh V

Ord T https://orcid.org/0000-0002-2608-2150

De Leo G

Portugal S https://orcid.org/0000-0002-2438-2352

Kirkman K

Nelson E

Girardin L

Azman A

Cox R

Lankau R

Cueva del Castillo R https://orcid.org/0000-0002-6385-0729

Secor WE

Pepke M

O'Shea RP https://orcid.org/0000-0003-3132-7199

Heise B

Blumberg S

Merckx V

Waldenström J

Ali J

Schlosser G

Scott-Samuel N

Arceo-Gomez G

Rees B https://orcid.org/0000-0001-5636-1700

Martin K

Ganusov V https://orcid.org/0000-0001-6572-1691

Manus M

Baker K https://orcid.org/0000-0003-3008-2513

Erten EY https://orcid.org/0000-0001-9736-0405

Wikström S

Eggleton P https://orcid.org/0000-0002-1420-7518

Liang W https://orcid.org/0000-0002-0004-9707

Peñalver E

Manzano-Marin A

Kraay A https://orcid.org/0000-0002-7042-7048

Campbell P https://orcid.org/0000-0001-7660-9814

Strader M

Chen X https://orcid.org/0000-0002-8164-3088

Linck E

Newsome T https://orcid.org/0000-0003-3457-3256

Fine P

Neate-Clegg M https://orcid.org/0000-0001-9753-6765

Clarke A

Patin E

Akcay C https://orcid.org/0000-0003-0635-9586

Tarwater C

Ottoni E https://orcid.org/0000-0003-1288-8345

Remacha Sebastian C https://orcid.org/0000-0002-3763-968X

Strode P

Lambers H

Lou N

Bujan J https://orcid.org/0000-0002-7938-0266

Stockley P https://orcid.org/0000-0003-1593-5848

Arnold T

Ratcliff W

Johnson R https://orcid.org/0000-0002-2431-0180

Moura R https://orcid.org/0000-0002-7911-4734

Simon R

Sarson G https://orcid.org/0000-0001-6774-9372

Troscianko J https://orcid.org/0000-0001-9071-2594

Sen B

Walmsley S https://orcid.org/0000-0001-8577-6884

Uricchio LH

Verma P

Rädecker N https://orcid.org/0000-0002-2387-8567

Zwoinska M

Bonduriansky R https://orcid.org/0000-0002-5786-6951

Tombak K https://orcid.org/0000-0002-7339-742X

Tedersoo L

Olea PP https://orcid.org/0000-0001-5387-9598

Maziarz M https://orcid.org/0000-0002-2921-5713

Reef R

Moland E

Alizon S https://orcid.org/0000-0002-0779-9543

Muscarella R

Pollock T https://orcid.org/0000-0001-5605-9069

Moe B https://orcid.org/0000-0002-2306-1899

Streipert S

Mollentze N

Close R https://orcid.org/0000-0003-3302-9902

Smith R

Caves E https://orcid.org/0000-0003-3497-5925

Hofmann H https://orcid.org/0000-0002-3335-330X

Harris R https://orcid.org/0000-0002-6227-5996

Zhao X

Prins H

Haaland T https://orcid.org/0000-0002-6968-4514

Raia P https://orcid.org/0000-0002-4593-8006

Soulié T https://orcid.org/0000-0002-6761-9196

Vorobyev M https://orcid.org/0000-0001-7615-5816

Horn A https://orcid.org/0000-0001-8850-6556

Grossnickle DM

Walker S

Abou Chakra M

Mainardi G https://orcid.org/0000-0003-4635-0586

Smolla M

Sepil I https://orcid.org/0000-0002-3228-5480

Novotny V https://orcid.org/0000-0001-7918-8023

Patlar B https://orcid.org/0000-0002-0442-9061

Metcalf C https://orcid.org/0000-0003-3166-7521

Caceres C

Potter C https://orcid.org/0000-0002-5223-8112

Walter G

Weston M https://orcid.org/0000-0002-8717-0410

Peignier M https://orcid.org/0000-0003-0849-4407

Wild G https://orcid.org/0000-0001-7821-7304

Shartau R https://orcid.org/0000-0002-0455-8496

Ruzicka F https://orcid.org/0000-0001-9089-624X

Podos J https://orcid.org/0000-0002-2546-7999

Frye M https://orcid.org/0000-0003-3277-3094

Cant M https://orcid.org/0000-0002-1530-3077

Samu F

Palacios O

Boulinier T

Rutschmann B https://orcid.org/0000-0001-6589-6408

Thacker C https://orcid.org/0000-0002-0700-734X

Queller D https://orcid.org/0000-0002-5464-1984

Ghalambor CK

Broggi J https://orcid.org/0000-0002-1706-4014

Lewis C https://orcid.org/0000-0002-3564-2906

Krupp DB https://orcid.org/0000-0001-6297-7739

Ceron K https://orcid.org/0000-0003-2354-3756

Lund A

Hsu C

Adams D

Fahimipour A

Dawson W

Weir J

Bengtson S

da Silva P https://orcid.org/0000-0002-0702-9186

Cosentino B https://orcid.org/0000-0002-6740-3133

Morgan T https://orcid.org/0000-0002-2848-8461

Suzuki M

Hartmann R

Pinkert S

Velicer G https://orcid.org/0000-0001-8721-1502

Krajcarz M https://orcid.org/0000-0002-1240-0664

Bisang I

Lotem A

Deacon A

Scott G https://orcid.org/0000-0002-4225-7475

Proches S

Geiselhardt S

Deutchman P

D'Aloia C

Antoniazzi R

Das S https://orcid.org/0000-0002-1597-1147

Radford C https://orcid.org/0000-0001-7949-9497

Vilhelmsen L https://orcid.org/0000-0002-5593-5722

Macartney E https://orcid.org/0000-0003-3866-143X

van Oppen M https://orcid.org/0000-0003-4607-0744

Eriksson K https://orcid.org/0000-0002-7164-0924

Nilsson L

Garant D https://orcid.org/0000-0002-8091-1044

Alston J https://orcid.org/0000-0001-5309-7625

Farnsworth A

Hufbauer R

Riva F https://orcid.org/0000-0002-1724-4293

Haig D

Immonen E https://orcid.org/0000-0003-1121-6950

Gómez P https://orcid.org/0000-0003-2830-4105

Pozzi A https://orcid.org/0000-0002-5640-2995

Paracchini S https://orcid.org/0000-0001-9934-8602

Deschamps M

Moreras A

Nemes C https://orcid.org/0000-0001-6910-5191

Van Belleghem S https://orcid.org/0000-0001-9399-1007

Krishnan A https://orcid.org/0000-0002-8317-4991

Ghalambor C

Hackett T

Heinicke M https://orcid.org/0000-0002-6021-8058

Rust J

Moiseff A

Yoris A

Strand C

Bardin J https://orcid.org/0000-0003-2382-4259

Wielgus E

Muller S

Burin G

Reijenga B https://orcid.org/0000-0003-4318-6987

Friedman J

Brook C https://orcid.org/0000-0003-4276-073X

Rader JA https://orcid.org/0000-0003-2419-3399

Favila ME

Cockburn A

Beechler B https://orcid.org/0000-0002-9711-4340

Kobayashi K https://orcid.org/0000-0002-9475-6807

Gardner A https://orcid.org/0000-0002-1304-3734

Qian S

Staicer C

Tamagnini D

Cortez M https://orcid.org/0000-0003-2555-7684

Zanoli A https://orcid.org/0000-0002-9402-5617

Vos M

Jacobs A

Burke N https://orcid.org/0000-0002-9843-926X

Toxopeus J

Avarguès-Weber A https://orcid.org/0000-0003-3160-6710

Wang L-Y https://orcid.org/0000-0001-6980-3782

Walker J https://orcid.org/0000-0002-9732-5738

Schakowski A

Taborsky M https://orcid.org/0000-0002-1357-4316

Bisconti M

Bourguignon T https://orcid.org/0000-0002-4035-8977

Longdon B https://orcid.org/0000-0001-6936-1697

Powers A https://orcid.org/0000-0002-0257-9488

Kubelka V

McDaniel S https://orcid.org/0000-0002-5435-7377

Fromhage L https://orcid.org/0000-0001-5560-6673

Perez-Barrales R

Shao R

Mutanen M

Perreault C https://orcid.org/0000-0002-3403-1943

Leung J https://orcid.org/0000-0001-5846-3401

Peng F https://orcid.org/0000-0002-1637-5611

Feldman M https://orcid.org/0000-0002-0664-3803

Rendall D https://orcid.org/0000-0001-5198-8766

Waltham NJ

Hilborn R

Riabinina O

Morris M

Filion A https://orcid.org/0000-0003-1198-3017

Galindo-Martínez CT https://orcid.org/0000-0001-5238-3099

Schiel D

Hill K

Saintilan N

Hardesty D

Jarrett B

Wilts B https://orcid.org/0000-0002-2727-7128

Brüniche-Olsen A

Pilakouta N https://orcid.org/0000-0001-8503-520X

Angielczyk K

Stark G

Doingo-Sananes M

Sansalone G

Summons R

Sarson G https://orcid.org/0000-0001-6774-9372

Laibl L

Soltis P https://orcid.org/0000-0001-9310-8659

Dyble M https://orcid.org/0000-0001-6861-1631

Carter G https://orcid.org/0000-0001-6933-5501

Tougeron K https://orcid.org/0000-0003-4897-3787

Ciccotto P https://orcid.org/0000-0002-0260-1964

McWilliams S https://orcid.org/0000-0002-9727-1151

Miller C

Rønsted N

Schaeffer P https://orcid.org/0000-0001-6230-8121

Friberg U https://orcid.org/0000-0001-6112-9586

Mitri S

Lo N http://orcid.org/0000-0003-2176-2840

Klug H https://orcid.org/0000-0003-3169-2452

Marden J

Cardoso M

Spencer H

Hoyle A

Haug C

Metcalfe N https://orcid.org/0000-0002-1970-9349

Dingwell J https://orcid.org/0000-0001-6990-4153

Roussel D https://orcid.org/0000-0002-8865-5428

Lehmann L https://orcid.org/0000-0001-9549-9577

Audino J

Hodson C

Baptista L https://orcid.org/0000-0002-4429-9855

Duchêne DA https://orcid.org/0000-0002-5479-1974

Dhawale A

Nowakowski J

Simons A https://orcid.org/0000-0002-0198-465X

Long T https://orcid.org/0000-0002-8708-2728

Zhang R

Carotenuto Y

Cant M https://orcid.org/0000-0002-1530-3077

Dong Y https://orcid.org/0000-0003-4550-2322

Fitzgerald E

Jeffries D https://orcid.org/0000-0003-1701-3978

Fritsch C https://orcid.org/0000-0003-2062-5799

Nelson D

Stinchcombe J

Peecook B

Arcese P

Manzini I

Murray G

Liu C https://orcid.org/0000-0002-8664-6931

Rouyer F

Bujan J https://orcid.org/0000-0002-7938-0266

Starkweather K https://orcid.org/0000-0002-1554-4567

Wignall A https://orcid.org/0000-0002-0152-8312

Xue BingKan https://orcid.org/0000-0003-0057-0268

Garcia M https://orcid.org/0000-0003-2014-7387

Farquharson K

Saadi A https://orcid.org/0000-0002-5113-0441

Borgerhoff Mulder M https://orcid.org/0000-0003-1117-5984

Thorogood R https://orcid.org/0000-0001-5010-2177

Yang Y

Gribble K http://orcid.org/0000-0002-8781-9523

Liu O https://orcid.org/0000-0002-9735-3384

Slate J https://orcid.org/0000-0003-3356-5123

Green D

Sgró C https://orcid.org/0000-0001-7950-2246

Kinney M

Rasher D

Ragonnet M

Russo L https://orcid.org/0000-0002-7343-9837

Conway B

Allen A

De Groote I https://orcid.org/0000-0002-9860-0180

van Klink R https://orcid.org/0000-0002-8125-1463

Pillay D https://orcid.org/0000-0002-9206-4794

Muscarella R

Lv L

Bhattacharya A

Baran N https://orcid.org/0000-0001-6270-0625

Deraje P

Reid J https://orcid.org/0000-0002-5007-7343

Ringler E

Caves E https://orcid.org/0000-0003-3497-5925

Hammerschmidt K

Hu B

Koehler F

Godoy P https://orcid.org/0000-0003-4519-5094

Herrmann M

Walsh M https://orcid.org/0000-0002-7517-2013

Bakasubramaniam K

Dunn P

Papaj D

Patterson S https://orcid.org/0000-0002-5283-8919

Williams S https://orcid.org/0000-0002-2251-2774

Ord T https://orcid.org/0000-0002-2608-2150

Wright D

Carey D

Nalla S

Gilbert A https://orcid.org/0000-0002-7632-8550

Graham Z https://orcid.org/0000-0002-6132-2885

Neate-Clegg M https://orcid.org/0000-0001-9753-6765

Pons L

Henshaw J https://orcid.org/0000-0001-7306-170X

Zahnd C

Luo M https://orcid.org/0000-0002-2975-5218

Title P

May S https://orcid.org/0000-0002-5312-140X

Kirschel A https://orcid.org/0000-0003-4379-7956

Falster D

Ritterbush K https://orcid.org/0000-0001-5604-7228

Bowen B

Potin G https://orcid.org/0009-0006-1004-3727

Haase M

Singer M

Olea PP. https://orcid.org/0000-0001-5387-9598

Correa RS

Sezmis A

Forester J

Gleiss A https://orcid.org/0000-0002-9960-2858

Delhey K https://orcid.org/0000-0001-5190-5406

Latorre D

Tandberg AH

Wang Y

Hector T

Savage A

Webb J

Huisman J https://orcid.org/0000-0002-1782-8109

Gilroy S

Oliver R

Fessler D

Foster-Dyer R https://orcid.org/0000-0001-9047-6851

Liu J https://orcid.org/0000-0001-6326-3180

Hsieh C-H

Jardine M

Huangfu C https://orcid.org/0000-0001-8369-7381

Kinoshita M https://orcid.org/0000-0002-5818-9622

Sadoughi B https://orcid.org/0000-0001-5626-3318

Alvarado F

Steffan-Dewenter I

Reed T

Ivimey-Cook E https://orcid.org/0000-0003-4910-0443

Bötzl F https://orcid.org/0000-0001-5121-3370

Lyal C

Lunau K https://orcid.org/0000-0001-5184-4201

Rondeau S https://orcid.org/0000-0002-5916-7441

Campana M

Simova I

Laudet V

Strode P

D'Aniello S https://orcid.org/0000-0001-7294-1465

Smit B

Hejnol A https://orcid.org/0000-0003-2196-8507

Beauchamp G https://orcid.org/0000-0002-3393-9562

Stockmaier S https://orcid.org/0000-0001-8280-8086

Restif O https://orcid.org/0000-0001-9158-853X

Leal CG https://orcid.org/0000-0002-0108-8572

De Roode J

Reeves A

Narendra A https://orcid.org/0000-0002-1286-5373

Capellini I

Álvarez-Presas M

Koneru M

Kampis D https://orcid.org/0000-0003-1678-3874

Grenzi M

Zaffos A

Beheim B

Remage-Healey L

Langergraber K

DuBose T

Gunderson A https://orcid.org/0000-0002-0120-4246

Arnold D https://orcid.org/0000-0003-1027-6222

Molho C https://orcid.org/0000-0002-2846-2544

Li S

Gomes S

DeRango E

Chippindale AK

Muotka T

Burraco P https://orcid.org/0000-0002-9007-2643

Jordal B

Marjakangas E-L https://orcid.org/0000-0002-5245-3779

Bretman A https://orcid.org/0000-0002-4421-3337

Champredon D https://orcid.org/0000-0002-7090-8757

Kort A

Brice E

Conti-Jerpe I

Cone R

Nakamura T https://orcid.org/0000-0003-0183-1685

Amsler C

Power D

Seymour R https://orcid.org/0000-0002-3395-0059

de Boer R

Herbertsson L https://orcid.org/0000-0002-8802-1409

Jones K

Kohl M

Mullon C https://orcid.org/0000-0002-9875-4227

Vasseur D

Ludt W

Seethapathi N https://orcid.org/0000-0002-5159-9717

Salmón P https://orcid.org/0000-0001-9718-6611

Ben Shaul Y

Drury J https://orcid.org/0000-0002-0262-8761

Morin O https://orcid.org/0000-0002-6216-1307

Perron G

Conant G https://orcid.org/0000-0002-8677-4933

Lee J

Emberts Z https://orcid.org/0000-0002-7949-0254

Mazo-Vargas A

Gokcumen O

Hetem R

Aspi J

westra edze https://orcid.org/0000-0003-4396-0354

Romano A https://orcid.org/0000-0002-7502-9268

Robert T https://orcid.org/0000-0002-6502-7082

Vannier J https://orcid.org/0000-0003-0998-1231

Frey ED

Tenger-Trolander A https://orcid.org/0000-0001-8319-0273

Strayer D

Barve S https://orcid.org/0000-0001-5840-8023

Perry M https://orcid.org/0000-0002-5977-8031

Hurst J

Kim S-Y https://orcid.org/0000-0002-5170-8477

Kantsa A

Trainor L https://orcid.org/0000-0003-3397-2079

Jani A https://orcid.org/0000-0001-8719-2962

Head M https://orcid.org/0000-0002-8123-7661

Liao J https://orcid.org/0000-0002-9520-3235

Iossa G https://orcid.org/0000-0001-6813-4361

Frith U https://orcid.org/0000-0002-9063-4466

Lichtenberg E

Kozłowski J https://orcid.org/0000-0002-7084-2030

Santos FP https://orcid.org/0000-0002-2310-6444

Patten M

Benowitz K https://orcid.org/0000-0002-7685-1827

Watts H https://orcid.org/0000-0002-9888-825X

Wang L https://orcid.org/0000-0002-2276-9529

Murray R https://orcid.org/0000-0001-6464-0072

Bradler S

Contador-Kelsall I

Moore J-S

Candidi M

Lin H-T

Lund A

Hare J

Bonduriansky R https://orcid.org/0000-0002-5786-6951

Kosmopoulos J

Pokorny T

Ward J https://orcid.org/0000-0001-7007-1902

Ekström A https://orcid.org/0000-0002-9966-8160

Bowles AMC https://orcid.org/0000-0002-7487-3811

Qian S

Parsch J

Macphie K

Baird E https://orcid.org/0000-0003-3625-3897

Belusic G https://orcid.org/0000-0003-3571-1948

Van Valkenburgh B

Sweet A https://orcid.org/0000-0003-2765-7021

Viana D

Dion E

Nehring V https://orcid.org/0000-0002-0494-1428

Ahrens D https://orcid.org/0000-0003-3524-7153

Bussière L

Lister A

Mongeau J-M https://orcid.org/0000-0002-3292-6911

Girard F https://orcid.org/0000-0002-7846-2710

Carmichael DA https://orcid.org/0000-0001-6938-1380

Lin Q

Streelman JT

Thorley J https://orcid.org/0000-0002-8426-610X

Bernardi G https://orcid.org/0000-0002-8249-4678

Chapuisat M https://orcid.org/0000-0001-7207-199X

Friedman N https://orcid.org/0000-0002-0533-6801

Fricker B

Larson WA

Antoniazzi M

Connallon T https://orcid.org/0000-0002-1962-4951

Araujo P

Raedecker N

Wolfner M

Wainwright P

Schmitz L https://orcid.org/0000-0003-0210-4383

Ridley A https://orcid.org/0000-0001-5886-0992

Okada Y https://orcid.org/0000-0003-4528-2980

Martinossi-Allibert I http://orcid.org/0000-0002-8332-8620

Saulnier-Talbot E

Dudley S https://orcid.org/0000-0002-8660-3720

Akimoto S-i https://orcid.org/0000-0002-7038-9678

Palme R

Dunn P

Wright W

Askew G https://orcid.org/0000-0003-1010-4439

Clark C https://orcid.org/0000-0001-7943-9291

Darcq E

Greenfield M https://orcid.org/0000-0003-1935-3423

Teder T https://orcid.org/0000-0001-6587-9325

Griesser M

Leng G https://orcid.org/0000-0002-2388-8466

Thomas G https://orcid.org/0000-0002-1982-6051

Karsdorp F https://orcid.org/0000-0002-5958-0551

Verberk W

Tarpy D

Koufopanou V

Dhungana A

Riehl C https://orcid.org/0000-0002-7872-336X

Anderson R

Hatta M

Dimitrov D https://orcid.org/0000-0001-5830-5702

Ambrosini R

García-Roger E

Mcilroy D https://orcid.org/0000-0001-9521-499X

Seamons T

Niemeier M

Miyazaki Y

Frederich B https://orcid.org/0000-0003-3438-0243

van Leeuwen J

Dukas R https://orcid.org/0000-0003-4533-8542

Orive M

McKechnie A https://orcid.org/0000-0002-1524-1021

Sibert E https://orcid.org/0000-0003-0577-864X

Perez-Tris J https://orcid.org/0000-0001-5535-3100

Willis K

Archie E https://orcid.org/0000-0002-1187-0998

Bleasdale B

Das B https://orcid.org/0000-0003-0855-262X

Immonen E https://orcid.org/0000-0003-1121-6950

Dufresne F https://orcid.org/0000-0002-2481-4864

Vasudeva R https://orcid.org/0000-0002-3831-0384

Martin J

Bourdeau PE

Delhey K https://orcid.org/0000-0001-5190-5406

Krishnan A https://orcid.org/0000-0002-8317-4991

Winternitz J

Raia P https://orcid.org/0000-0002-4593-8006

Gill R https://orcid.org/0000-0001-9389-1284

van den Berg P https://orcid.org/0000-0001-7886-9345

Silber K

Milet-Pinheiro P

Evans S https://orcid.org/0000-0001-5654-8495

Hecht E

Immonen E https://orcid.org/0000-0003-1121-6950

Maiorano L

Li Y

van Heerwaarden B https://orcid.org/0000-0003-2435-2900

Kendall C https://orcid.org/0000-0003-4429-4496

Walmsley S https://orcid.org/0000-0001-8577-6884

Christie M https://orcid.org/0000-0001-7285-5364

Andre G

Rudman S

Hansen W

García-Algarra J

Orr P

Rimbach R https://orcid.org/0000-0003-3059-0382

Braem S https://orcid.org/0000-0002-9520-9741

Regan C https://orcid.org/0000-0002-5458-5687

Henshaw J https://orcid.org/0000-0001-7306-170X

Emer C

Limburg K https://orcid.org/0000-0003-3716-8555

Tibblin P https://orcid.org/0000-0001-6804-5342

Meiklejohn C

Benson JF

Gehman A-L https://orcid.org/0000-0003-0595-9950

Ward J https://orcid.org/0000-0001-7007-1902

Ferrari M

Olsson M https://orcid.org/0000-0002-4130-1323

McHenry M https://orcid.org/0000-0001-5834-674X

Flack A

Bribiesca-Contreras G

Kellermann J

Seddon P

Buchanan K

Hudson C

Lukas D https://orcid.org/0000-0002-7141-3545

Hood W https://orcid.org/0000-0002-8398-3908

Leibold M

Plumlee J https://orcid.org/0000-0002-0411-5919

Fassbinder-Orth C

Minamoto T

Morris W

Aguirre JD

Prochazka P https://orcid.org/0000-0001-9385-4547

Takahashi A

Szathmary E https://orcid.org/0000-0001-5227-2997

Vontas J https://orcid.org/0000-0002-8704-2574

De Leo G

Ng WH https://orcid.org/0000-0002-5738-4960

Wersebe M

Abernathy V

Iwaniuk A https://orcid.org/0000-0001-9273-3655

Romano A https://orcid.org/0000-0002-0945-6018

Langangen Ø

Nourani E https://orcid.org/0000-0003-4420-3902

Kendall L https://orcid.org/0000-0002-0671-0121

Hughes A https://orcid.org/0000-0003-2677-1965

Wagner P

Ślusarczyk M https://orcid.org/0000-0002-2311-5033

Hetem R

Kratochwil C https://orcid.org/0000-0002-5646-3114

Reinhardt J https://orcid.org/0000-0002-1345-2976

Di Bernardi C

Bogo G

Malcolm S

Penacchio O https://orcid.org/0000-0002-1544-2405

Chang C-H https://orcid.org/0000-0001-9361-1190

Belyaev R

Cronberg N

Rose N https://orcid.org/0000-0001-7129-4753

Ramírez-Castañeda V

Wilfert L https://orcid.org/0000-0002-6075-458X

Trifonov V https://orcid.org/0000-0003-0454-8359

Karatayev V https://orcid.org/0000-0001-7112-8069

Pola M

Shawkey M https://orcid.org/0000-0002-5131-8209

Stibor H

Blanco F https://orcid.org/0000-0001-6339-3460

Allen CN

Reed T

Barott K https://orcid.org/0000-0001-7371-4870

Podos J https://orcid.org/0000-0002-2546-7999

Street G https://orcid.org/0000-0002-1260-9214

Enfield NJ https://orcid.org/0000-0003-3891-6973

Merchant H

Griffiths J https://orcid.org/0000-0003-0319-515X

Muylaert R https://orcid.org/0000-0002-6466-6210

Ostrowski E https://orcid.org/0000-0002-9784-5979

Leu ST

Ro T

Sackton T http://orcid.org/0000-0003-1673-9216

Visser M https://orcid.org/0000-0002-1456-1939

Fristoe T

Morris M

Clark N https://orcid.org/0000-0001-7131-3301

Shimeld S https://orcid.org/0000-0003-0195-7536

Haase M

Carotenuto Y

Jasienska G https://orcid.org/0000-0001-8716-6342

Chazdon RL

Spagna J

Favilla A https://orcid.org/0000-0002-6370-0499

Smith J

Mahoney S https://orcid.org/0000-0001-8446-423X

Darroch S https://orcid.org/0000-0003-1922-7136

Smith O

Sexton J

Hunt B

Lotem A

Olea PP https://orcid.org/0000-0001-5387-9598

Nikelski E

Fryxell J https://orcid.org/0000-0002-5278-8747

Bonser S

Dahlgren J http://orcid.org/0000-0002-1125-9769

Wang Y

Allen B https://orcid.org/0000-0003-0282-6407

Rubio De Casas R http://orcid.org/0000-0003-4276-4968

Wilcox TM

Fine M https://orcid.org/0000-0001-9665-1462

Lehmann G

Jernvall J https://orcid.org/0000-0001-6575-8486

Royle N

Lynn A

Bolnick D https://orcid.org/0000-0003-3148-6296

Kobayashi K https://orcid.org/0000-0002-9475-6807

Cairns J https://orcid.org/0000-0003-1329-2025

Michels E

Chen L https://orcid.org/0000-0002-0394-4387

Pyenson N https://orcid.org/0000-0003-4678-5782

Prokopenko C

Swedo J

Burns K https://orcid.org/0000-0002-4938-2877

Moles A https://orcid.org/0000-0003-2041-7762

Yamamichi M https://orcid.org/0000-0003-2136-3399

Miller J

Yi X https://orcid.org/0000-0003-4860-7429

Matesanz S https://orcid.org/0000-0003-0060-6136

Villinger J

Fugere V

Tehrani J

Ross S https://orcid.org/0000-0003-3512-9579

Christofferson R https://orcid.org/0000-0003-2806-1131

Strand C

Garcia-R J https://orcid.org/0000-0002-6665-9671

Brand J https://orcid.org/0000-0003-3312-941X

Olsson M https://orcid.org/0000-0002-4130-1323

Tennant W https://orcid.org/0000-0001-5822-6887

Fischer E https://orcid.org/0000-0002-2916-0900

terHorst C

Balakrishnan R

Pirk C

Fromm B

Schakowski A

Richardson J https://orcid.org/0000-0001-5839-9315

Moles J

Shultz A

Andruchow Colombo A

Sharma N https://orcid.org/0000-0002-7411-5594

Titcomb G

Feng J https://orcid.org/0000-0002-7503-1069

Inoue W

McNickle G https://orcid.org/0000-0002-7188-7265

Broggi J https://orcid.org/0000-0002-1706-4014

Verberk W

Nakamura T https://orcid.org/0000-0003-0183-1685

Wang Y https://orcid.org/0000-0003-3011-1919

Błaszczyk A

Raoult V https://orcid.org/0000-0001-9459-111X

Summers K https://orcid.org/0000-0001-7292-4286

Vannette R https://orcid.org/0000-0002-0447-3468

Baptista L https://orcid.org/0000-0002-4429-9855

Luis Rodriguez J

Davis A

Bury S

Kellermann V

Feyrer L https://orcid.org/0000-0002-9300-1947

Kristensen J

Oro D https://orcid.org/0000-0003-4782-3007

Monaghan P https://orcid.org/0000-0003-2430-0326

Noguer Calabus I

Carazo P https://orcid.org/0000-0002-1525-6522

Theobald J

Powers A https://orcid.org/0000-0002-0257-9488

Oliveira B

Mashoodh R

Wild G https://orcid.org/0000-0001-7821-7304

Foellmer M https://orcid.org/0000-0003-2331-9754

Fordyce J

Streit R

Kelley J https://orcid.org/0000-0002-8223-7241

Lomolino M

Barnett J https://orcid.org/0000-0001-9789-4132

Patel R https://orcid.org/0000-0002-5323-2062

Arnott S

Pathak A

Neumann C

Ratcliff W

Cusimano M https://orcid.org/0000-0002-7435-2434

Kniss A

Purcell J

Goodbody-Gringley G

Fischer J https://orcid.org/0000-0002-5807-0074

Nowicki P

Tariel-Adam J https://orcid.org/0000-0003-1552-4088

Sackville M https://orcid.org/0000-0001-8206-6836

Cortez M https://orcid.org/0000-0003-2555-7684

Gasparini C https://orcid.org/0000-0001-9172-1142

Joergensen L

Minias P https://orcid.org/0000-0002-7742-6750

Senderecka M

Horn A https://orcid.org/0000-0001-8850-6556

Furuichi T

Battaglini L

Collet J https://orcid.org/0000-0002-6028-5234

Hemmi J https://orcid.org/0000-0003-4629-9362

Baskett M

Karve S

Rokitta S

Wong MKL https://orcid.org/0000-0002-6248-3103

Wegner KMs https://orcid.org/0000-0002-2410-8898

Pataky T

Capelli C

Clements H

Freeberg T https://orcid.org/0000-0003-3807-4147

McLean C

Giachetti C

Glastad K

Hettyey A

D'Aloia C

Goulet T https://orcid.org/0000-0003-3883-6543

Atmore L

Richardson D

Franklin A

Shipley O https://orcid.org/0000-0001-7533-5620

Stinchcombe J

Hörnig MK

Wylie D

Watts D https://orcid.org/0000-0001-6603-2230

Vitousek M https://orcid.org/0000-0003-2855-0832

Leung T

Elgar M https://orcid.org/0000-0003-0861-6064

Aich U

Somanathan H https://orcid.org/0000-0002-4803-3894

Vargas A

Pereira R https://orcid.org/0000-0002-8076-4822

McAfee D https://orcid.org/0000-0001-8278-8169

Lyamin O https://orcid.org/0000-0003-2049-0943

Briedis M

Lev Ari S

Chakarov N

Traniello I

Shepard E

Renner S https://orcid.org/0000-0003-3704-0703

Li-Byarlay H https://orcid.org/0000-0001-6579-5172

DuVal E

Guentuerkuen O

Bodawatta K https://orcid.org/0000-0002-6095-9059

Spoelstra K https://orcid.org/0000-0001-8614-4387

Challis J

Hufbauer R

Fiedler W

Ahlberg P https://orcid.org/0000-0001-9054-2900

Rasmussen J

Harris R https://orcid.org/0000-0002-6227-5996

Rajabi H

Monroy J

Phillips J

Belusic G https://orcid.org/0000-0003-3571-1948

Rasotto M https://orcid.org/0000-0001-5711-2810

Simons M https://orcid.org/0000-0001-7406-7708

Caro T https://orcid.org/0000-0001-6804-8519

Wang R https://orcid.org/0000-0003-4652-2149

Vellwock A

Boots M https://orcid.org/0000-0003-3763-6136

Raymond B https://orcid.org/0000-0002-3730-0985

Hemmings N https://orcid.org/0000-0003-2418-3625

Amoroso C https://orcid.org/0000-0001-5456-7975

Moser C https://orcid.org/0000-0002-5068-0740

Todorov O https://orcid.org/0000-0002-0295-7557

Merino S

Carrier T https://orcid.org/0000-0001-7885-184X

Arnqvist G https://orcid.org/0000-0002-3501-3376

Mollon J https://orcid.org/0000-0001-8533-033X

Hovel K

Harborne A

Suarez C

Havens H

Hopkins S

Luo J

Arnqvist G https://orcid.org/0000-0002-3501-3376

McIntosh A

Yvon-Durocher G

Blakeslee A https://orcid.org/0000-0001-9667-2175

Perrot-Minnot M-J https://orcid.org/0000-0003-3412-4282

Duguid S

Leleu A https://orcid.org/0000-0002-2943-8183

Luhring T https://orcid.org/0000-0001-7982-5862

Mongue A

Broitman B https://orcid.org/0000-0001-6582-3188

Jaakkola K https://orcid.org/0000-0002-4113-748X

Endler J https://orcid.org/0000-0002-7557-7627

Boukal D https://orcid.org/0000-0001-8181-7458

Kim S-Y https://orcid.org/0000-0002-5170-8477

Narbona E

Kitano J https://orcid.org/0000-0001-8659-5698

Godoy P https://orcid.org/0000-0003-4519-5094

Härer A https://orcid.org/0000-0003-2894-5041

Glaser-Schmitt A

Monks A

Greenfield M https://orcid.org/0000-0003-1935-3423

Lynch K

Tejedo M

Sulser R https://orcid.org/0000-0002-8750-0942

Rolland J

Harrison R

Kirschel A https://orcid.org/0000-0003-4379-7956

Evans S

Gerson A

Forde T

Kunz J

Willett C

Mazzocchi MG

Trifonov V https://orcid.org/0000-0003-0454-8359

Hindar K

Fichtel C https://orcid.org/0000-0002-8346-2168

Paniw M https://orcid.org/0000-0002-1949-4448

Pascalis O

Zimmer A

Davies S

De Baets K https://orcid.org/0000-0002-1651-321X

Potter C https://orcid.org/0000-0002-5223-8112

Charlat S

Pons L

Domínguez Guerrero S

Ludsin S https://orcid.org/0000-0002-3866-2216

Hobson E

Balfour N https://orcid.org/0000-0002-2426-3898

Villinger J

Brivio F https://orcid.org/0000-0002-1449-8335

Bertram J

Parker B

Benenson JF

Lacoursiere A

Tye S

Futahashi R

Beardall J

Santos Matos G

Morrison C https://orcid.org/0000-0002-4293-2717

Guenther A https://orcid.org/0000-0002-2350-0185

Hanus R

Sun S-J https://orcid.org/0000-0002-7859-9346

Heard SB

Witt C https://orcid.org/0000-0003-2781-1543

Dong Y https://orcid.org/0000-0003-4550-2322

Rodríguez R https://orcid.org/0000-0003-0262-0839

Hughes N

Zuo R

Allen B https://orcid.org/0000-0003-0282-6407

Haux L

Hector T

Briolat E https://orcid.org/0000-0001-5695-1065

Bicknell R https://orcid.org/0000-0001-8541-9035

Donelan S https://orcid.org/0000-0002-4066-7884

Lupyan G https://orcid.org/0000-0001-8441-7433

Tekwa E https://orcid.org/0000-0003-2971-6128

Bendrey R

Axelrod C https://orcid.org/0000-0002-6130-0168

Amiri E https://orcid.org/0000-0001-8536-5815

Gleiss A https://orcid.org/0000-0002-9960-2858

Becciu P https://orcid.org/0000-0003-2145-6667

Santos E https://orcid.org/0000-0003-3158-7935

Friberg U https://orcid.org/0000-0001-6112-9586

Dyble M https://orcid.org/0000-0001-6861-1631

Silvestro D

Råberg L https://orcid.org/0000-0001-5219-7448

Kissane R https://orcid.org/0000-0001-9385-2584

Slade J

Barreto E https://orcid.org/0000-0002-3372-7295

Nol E

Rebora M https://orcid.org/0000-0002-4271-6336

Garcia DS

Gebauer G

Moiseff A

Rodgers K

Ross P

Bairos-Novak K https://orcid.org/0000-0002-0152-1452

Michell C

Volckaert F

Landler L https://orcid.org/0000-0002-5638-7924

Mensinger A

Nehring V https://orcid.org/0000-0002-0494-1428

Smith R

Ferrante M

Šobotník J https://orcid.org/0000-0002-8581-637X

Richardson D https://orcid.org/0000-0001-7226-9074

Verberk W

Pardi M https://orcid.org/0000-0001-9766-7511

Ma L

Glasel H

Twiname S

Lavanchy G

Oppel S

Eggleton P https://orcid.org/0000-0002-1420-7518

Ims R

Anikin A https://orcid.org/0000-0002-1250-8261

Gardner A https://orcid.org/0000-0002-1304-3734

Sudyka J

Thorogood R https://orcid.org/0000-0001-5010-2177

DeSiervo M

Chikaraishi Y

Nebauer C

Lazzaro B https://orcid.org/0000-0002-3881-0995

Guevara-Fiore P http://orcid.org/0000-0003-2222-3777

Vicari D

Michelangeli M

Arrighi R https://orcid.org/0000-0002-5435-6729

Dolan R

Rousselle M http://orcid.org/0000-0003-3930-0732

Genner M https://orcid.org/0000-0003-1117-9168

Kobylkov D

Walker J https://orcid.org/0000-0002-9732-5738

Martínez-Vilalta J

Gil M

Kulahci I https://orcid.org/0000-0003-0104-0365

Conti-Jerpe I

Askew G https://orcid.org/0000-0003-1010-4439

Wascher C

Martel SI https://orcid.org/0000-0002-7469-6878

Czuppon P https://orcid.org/0000-0003-1462-7237

Morsky B

Simone-Finstrom M https://orcid.org/0000-0003-2938-9788

Hovick S

Keith D

Pomiankowski A https://orcid.org/0000-0002-5171-8755

Gottdenker N

Watanabe A https://orcid.org/0000-0001-5057-4772

Morimoto J https://orcid.org/0000-0003-3561-1920

Datta A

Dunhill A https://orcid.org/0000-0002-8680-9163

Abbatecola C

Mindlin G

Hastings I

Barclay K

Lyu S https://orcid.org/0000-0003-0221-5315

Gardner J

Herencias C

Sauer F

Bouyoucos I https://orcid.org/0000-0002-4267-1043

Hitchcock T https://orcid.org/0000-0002-6378-5023

Leimar O https://orcid.org/0000-0001-8621-6977

Molleman L https://orcid.org/0000-0001-7978-7173

cai wang https://orcid.org/0000-0002-8132-5821

Martin J https://orcid.org/0000-0001-8704-6076

LaMontagne J https://orcid.org/0000-0001-7713-8591

Warne R https://orcid.org/0000-0001-7568-074X

Lucas J https://orcid.org/0000-0001-9661-7985

Griffith S https://orcid.org/0000-0001-7612-4999

Pyenson N https://orcid.org/0000-0003-4678-5782

Iwaniuk A https://orcid.org/0000-0001-9273-3655

Matthews B

Veller C

García-Roger E

Elston D

Greenwood J https://orcid.org/0000-0002-6184-0818

Höner O

Tennant W https://orcid.org/0000-0001-5822-6887

Getty T

Merckx V

Wojczulanis-Jakubas K

Smith J https://orcid.org/0000-0003-4633-4519

Michels E

Dreissig S https://orcid.org/0000-0002-4766-9698

Faust CL https://orcid.org/0000-0002-8824-7424

Kwon E

Gómez-Bahamón V

Krueger-Hadfield S

Burns S https://orcid.org/0000-0001-8695-3643

Lin D

Manzano-Marin A

Baldwin M

Volodin I https://orcid.org/0000-0001-6278-0354

Breed G

Reis C

Lyu L

Grueter C https://orcid.org/0000-0001-8770-8148

Moffett E https://orcid.org/0000-0001-9891-6217

Sanmartín-Villar I https://orcid.org/0000-0002-5008-7613

Osada N https://orcid.org/0000-0003-0180-5372

Kubelka V

Figueroa L https://orcid.org/0000-0003-0655-8278

Flintham E https://orcid.org/0000-0002-8037-5560

Weisbecker V https://orcid.org/0000-0003-2370-4046

Selier SAJ

Dimitrov D https://orcid.org/0000-0001-5830-5702

Raposo do Amaral F https://orcid.org/0000-0002-1133-3789

Jablonski D

Morea M

Stothart M https://orcid.org/0000-0002-2863-908X

Herniou E

Heathcote R

Naug D https://orcid.org/0000-0001-9252-8037

Moeller A

Matassa C https://orcid.org/0000-0003-2632-6191

Butts I

Starkweather K https://orcid.org/0000-0002-1554-4567

Schneider J https://orcid.org/0000-0001-8523-7354

Pisanski K https://orcid.org/0000-0003-0992-2477

Reichard M https://orcid.org/0000-0002-9306-0074

Mizumoto N https://orcid.org/0000-0002-6731-8684

Seger J

Foley A

Lynn A

Wheeler C

Demartsev V https://orcid.org/0000-0002-1456-9789

Ince R

Korb J https://orcid.org/0000-0001-9577-9376

Cresswell W https://orcid.org/0000-0002-4684-7624

Roeder K https://orcid.org/0000-0002-2628-5003

Christofferson R https://orcid.org/0000-0003-2806-1131

Turetzek N

McGechie F

Üveges B https://orcid.org/0000-0001-9234-9258

Mysterud A https://orcid.org/0000-0001-8993-7382

Schmid D

Hinde K

Shutler D

Ivimey-Cook E https://orcid.org/0000-0003-4910-0443

Rossi N

Stockmaier S https://orcid.org/0000-0001-8280-8086

Yang L

Manassi M https://orcid.org/0000-0003-4210-7570

Audet J-Nicolas

Gershman S https://orcid.org/0000-0003-4451-820X

Rasmussen J

Houdelier C

Michaud M https://orcid.org/0000-0001-7075-9862

Title P

Gogarten J https://orcid.org/0000-0003-1889-4113

Ottaviani G https://orcid.org/0000-0003-3027-4638

Sol D https://orcid.org/0000-0001-6346-6949

Hernández-Gómez O

Philson C https://orcid.org/0000-0002-5974-347X

Lucon-Xiccato T https://orcid.org/0000-0002-1017-9230

Berumen M https://orcid.org/0000-0003-2463-2742

Thomas L https://orcid.org/0000-0002-7436-067X

Visser M https://orcid.org/0000-0002-1456-1939

Corsini M https://orcid.org/0000-0001-5196-086X

Beaudet A https://orcid.org/0000-0002-9363-5966

Antony J

Lasky J https://orcid.org/0000-0001-7688-5296

Alvarez-Filip L https://orcid.org/0000-0002-5726-7238

Dujon A https://orcid.org/0000-0002-1579-9156

Rondinini C

Trifonov V https://orcid.org/0000-0003-0454-8359

Spaak J https://orcid.org/0000-0001-5157-9188

Mainardi G https://orcid.org/0000-0003-4635-0586

Luhring T https://orcid.org/0000-0001-7982-5862

James L https://orcid.org/0000-0002-9600-4473

Goss-Souza D

Forde T

Wallace M

Albery G https://orcid.org/0000-0001-6260-2662

Hansen W

Miller A

Kellermann V

Roth O https://orcid.org/0000-0002-7349-7797

Sørensen M

Hacket-Pain A https://orcid.org/0000-0003-3676-1568

Ito K https://orcid.org/0000-0002-1470-3677

Ammar M

Szabo B https://orcid.org/0000-0002-3226-8621

Pilakouta N https://orcid.org/0000-0001-8503-520X

Margiotta-Casaluci L

Russo L https://orcid.org/0000-0002-7343-9837

Chevin L-Miguel https://orcid.org/0000-0003-4188-4618

Ghedini G https://orcid.org/0000-0002-5156-2009

Joyce T

Teixeira J

Hettyey A

Lund A

Wendling C https://orcid.org/0000-0003-4532-7528

Pierce S

Girard-Buttoz C https://orcid.org/0000-0003-1742-4400

Davidowitz G

Gethmann J

Pratt S https://orcid.org/0000-0002-1086-4019

Wuttke M

Miyashita T

Henrique D

Pizo M

Miles D

Capellini I

Rubio De Casas R http://orcid.org/0000-0003-4276-4968

Contemori G https://orcid.org/0000-0002-1873-1472

Rutschmann B https://orcid.org/0000-0001-6589-6408

Mori E

Chudzinska M

Perez D https://orcid.org/0000-0002-8560-0368

Barrett R

Azevedo R

Branco S

Ross P

Ringler E

Szathmary E https://orcid.org/0000-0001-5227-2997

Härer A https://orcid.org/0000-0003-2894-5041

Ratcliff W

Sheehan M https://orcid.org/0000-0002-3949-7873

Gururaja KV

Sowersby W https://orcid.org/0000-0002-9774-5935

Bruijn S https://orcid.org/0000-0003-0290-2131

Wang P

Wang B https://orcid.org/0000-0003-1010-6573

Brander K

Oswald K https://orcid.org/0000-0001-8745-4432

Shah S

Ross C https://orcid.org/0000-0001-7764-761X

Mullon C https://orcid.org/0000-0002-9875-4227

Reschechtko S

Oliver K

Yoshioka Y

Millien V https://orcid.org/0000-0002-1390-766X

Castañeda LE https://orcid.org/0000-0001-5484-4573

Hitchcock T https://orcid.org/0000-0002-6378-5023

Pirk C

Ferrante M

Newbury A

Chelo I

Sugiura S https://orcid.org/0000-0002-9655-2189

Pardo J https://orcid.org/0000-0002-2665-8893

Burczyk J

Goodisman M https://orcid.org/0000-0002-4842-3956

Ackerley R https://orcid.org/0000-0003-4621-7929

Lahiff N

Bellvert A

Banisch S

Swedo J

Elgar M https://orcid.org/0000-0003-0861-6064

Hörnig MK

Brand J https://orcid.org/0000-0003-3312-941X

Cortez M https://orcid.org/0000-0003-2555-7684

Li S

Reijenga B https://orcid.org/0000-0003-4318-6987

Pebsworth P

Keyser S

Helantera H https://orcid.org/0000-0002-6468-5956

Rohner P https://orcid.org/0000-0002-9840-1050

Ocampo D https://orcid.org/0000-0002-7411-2027

Strauss E https://orcid.org/0000-0003-3413-1642

Jeffery K https://orcid.org/0000-0002-2632-0008

Yi X https://orcid.org/0000-0003-4860-7429

Hay M

Dreujou E

Ayala Lara R https://orcid.org/0000-0002-3239-0854

Rozen-Rechels D

Dong S-L

Makin A https://orcid.org/0000-0002-4490-7400

Hesse E https://orcid.org/0000-0002-1900-7136

Solan M https://orcid.org/0000-0001-9924-5574

M. Silva LG https://orcid.org/0000-0002-2329-5601

De Leo G

McLaren JD

Deutchman P

Koch E

Harvey T https://orcid.org/0000-0002-2717-7004

Zayed A https://orcid.org/0000-0003-3233-4585

Pinto-Ledezma JN https://orcid.org/0000-0001-6668-9670

Duchenne F https://orcid.org/0000-0002-6917-8013

Olsson M https://orcid.org/0000-0002-4130-1323

Farrell T https://orcid.org/0000-0002-5005-3536

Chakraborty S

Moyle L

Reudink M

Matheus J

Kominsky JFF

Scacco M

Sanders D https://orcid.org/0000-0003-2383-8693

Toussaint EFA

Dijkerman C https://orcid.org/0000-0002-5364-3312

Fisher D

Kokko H https://orcid.org/0000-0002-5772-4881

Petrullo L https://orcid.org/0000-0001-6464-3177

Halsey L https://orcid.org/0000-0002-0786-7585

Ruffley M

Lima M https://orcid.org/0000-0002-3700-2945

Malcolm S

Cohen J https://orcid.org/0000-0001-9611-9150

Udekwu K

Ord T https://orcid.org/0000-0002-2608-2150

Alizon S https://orcid.org/0000-0002-0779-9543

Armstrong E

Dalle Fratte M

Hurst L https://orcid.org/0000-0002-1002-1054

Schultheiss P https://orcid.org/0000-0002-6906-3507

Schwarz S https://orcid.org/0000-0002-6327-6181

Xie G https://orcid.org/0000-0002-7125-7386

de Angeli Dutra D https://orcid.org/0000-0003-2341-2035

Deacon A

Page M

Griffith S https://orcid.org/0000-0001-7612-4999

Arikawa K https://orcid.org/0000-0002-4365-0762

Anton S

Herben T

Liao J https://orcid.org/0000-0002-9520-3235

Hoyle A

Barona-Gomez F

Fernández Domingos E https://orcid.org/0000-0002-4717-7984

Filipiak Z

Moehring A https://orcid.org/0000-0002-8088-4007

Wishart A https://orcid.org/0000-0001-7954-0613

Shoji A https://orcid.org/0000-0003-3364-7786

Ye M

Pisor A

Hudson C

Steinmann T https://orcid.org/0000-0002-2013-7747

Sin SYW

Owens H https://orcid.org/0000-0003-0071-1745

Fristoe T

Titcomb G

Asgari D

Karban R https://orcid.org/0000-0002-1565-2429

Wang L https://orcid.org/0000-0002-2276-9529

Prakash V

Johnson P https://orcid.org/0000-0001-6087-7064

Calhim S https://orcid.org/0000-0001-9059-2641

Gilman R https://orcid.org/0000-0003-2000-7966

Amos W https://orcid.org/0000-0002-0971-9914

Senderecka M

Humphrey S

Berger D https://orcid.org/0000-0003-0196-6109

McIntyre T https://orcid.org/0000-0001-8395-7550

Martins K

Tarwater C

DiSanto V

Orio A https://orcid.org/0000-0002-5566-6139

Aich U

Maglianesi M https://orcid.org/0000-0002-4053-6956

cardini andrea https://orcid.org/0000-0003-2910-632X

Bernal M

Poulin R https://orcid.org/0000-0003-1390-1206

Guentuerkuen O

Collins S

Martin-Duran J https://orcid.org/0000-0002-2572-1061

Ribak G https://orcid.org/0000-0002-6267-5471

McNally L

McCullough E https://orcid.org/0000-0002-6886-9445

Tinius A

Smith R

Crowley-Gall A https://orcid.org/0000-0003-2351-7267

Holyoak M

Saulnier-Talbot E

Kostal V https://orcid.org/0000-0002-4994-5123

Felice RN

Gómez P https://orcid.org/0000-0003-2830-4105

Meuti M https://orcid.org/0000-0003-4607-1392

Van Goor J https://orcid.org/0000-0001-7498-9315

Fordyce J

Preuss A https://orcid.org/0000-0003-0811-1613

Hedrick T https://orcid.org/0000-0002-6573-9602

Roney J https://orcid.org/0000-0002-5389-383X

Porto L https://orcid.org/0000-0001-8862-9839

Sheinkopf SJ

Albo MJ https://orcid.org/0000-0003-4332-7612

Hermes-Lima M

Mérot C https://orcid.org/0000-0003-2607-7818

Weinberger V https://orcid.org/0000-0002-9693-7492

Scanlan P https://orcid.org/0000-0002-6733-9653

Darroch S https://orcid.org/0000-0003-1922-7136

Garcia-Gonzalez F https://orcid.org/0000-0001-9515-9038

Henshaw J https://orcid.org/0000-0001-7306-170X

Römer H https://orcid.org/0000-0003-4326-0470

Thoré E

Killam D https://orcid.org/0000-0001-7569-1828

Kim S-Y https://orcid.org/0000-0002-5170-8477

Stiller J https://orcid.org/0000-0001-6009-9581

Wang R https://orcid.org/0000-0003-4652-2149

Ghalambor CK

Tidau S https://orcid.org/0000-0003-0336-0450

Duguid S

Samuels J

Benestan L https://orcid.org/0000-0003-1638-9893

Miyazaki Y

Beckerman A

Lattorff M https://orcid.org/0000-0002-8603-6332

Harvey C https://orcid.org/0000-0002-2830-0844

Karatayev V https://orcid.org/0000-0001-7112-8069

Silverman A

Battaglini L

Heubel K

Dueñas L https://orcid.org/0000-0002-0756-826X

Makin A https://orcid.org/0000-0002-4490-7400

Crevecoeur F

Teitelbaum C https://orcid.org/0000-0001-5646-3184

van Heerwaarden B https://orcid.org/0000-0003-2435-2900

Récapet C https://orcid.org/0000-0001-5414-8412

Helantera H https://orcid.org/0000-0002-6468-5956

Mutanen M

Tomášek O https://orcid.org/0000-0002-2657-5916

Armitage A

van Breugal F

Clifton G

Glazier D https://orcid.org/0000-0001-7164-1823

Daversa D https://orcid.org/0000-0002-8984-8897

Sexton J

Menezes J https://orcid.org/0000-0003-4156-2934

Püschel T

Marrec L https://orcid.org/0000-0003-0941-6603

Rong Z

Paraskevopoulou S https://orcid.org/0000-0003-4274-0579

Fisher J

Maia A

Tompros A

Fukuda Y

Ruzicka F https://orcid.org/0000-0001-9089-624X

Lane P

Lou N

Hamer G https://orcid.org/0000-0002-9829-788X

Cooper N https://orcid.org/0000-0002-4667-1542

Wang HaiJun https://orcid.org/0000-0001-6963-2557

Dang X https://orcid.org/0000-0002-1035-1600

Ke H-M

Contemori G https://orcid.org/0000-0002-1873-1472

Danise S https://orcid.org/0000-0002-6098-609X

Gibson A https://orcid.org/0000-0002-0867-4953

Jensen O https://orcid.org/0000-0003-0172-6578

Reynolds S https://orcid.org/0000-0001-5043-0242

Gilbert A

Denison F

Yang Q

Bonato M

Burke N https://orcid.org/0000-0002-9843-926X

Smith O

Thompson González N

Deng K https://orcid.org/0000-0003-2500-6794

Bernardi G https://orcid.org/0000-0002-8249-4678

Field J https://orcid.org/0000-0003-0663-4031

Slade J

Wagner P

